# Wastewater-powered high-value chemical synthesis in a hybrid bioelectrochemical system

**DOI:** 10.1016/j.isci.2021.103401

**Published:** 2021-11-06

**Authors:** Ranran Wu, Yang-Yang Yu, Yuanming Wang, Yan-Zhai Wang, Haiyan Song, Chunling Ma, Ge Qu, Chun You, Zhoutong Sun, Wuyuan Zhang, Aitao Li, Chang Ming Li, Yang-Chun Yong, Zhiguang Zhu

**Affiliations:** 1 Tianjin Institute of Industrial Biotechnology, Chinese Academy of Sciences, 32 West 7th Avenue, Tianjin Airport Economic Area, Tianjin 300308, P.R. China; 2 Biofuels Institute, School of the Environment and Safety Engineering, Jiangsu University, 301 Xuefu Road, Zhenjiang 212013, P.R. China; 3 University of Chinese Academy of Sciences, 19A Yuquan Road, Shijingshan District, Beijing, 100049, P.R. China; 4 State Key Laboratory of Biocatalysis and Enzyme Engineering, School of Life Sciences, Hubei University, Wuhan, 430062, P. R. China; 5 Institute for Materials Science and Devices, School of Materials Science and Engineering, Suzhou University of Science and Technology, Suzhou 215011, P.R. China; 6 Institute of Advanced Cross-Field Science, College of Life Sciences, Qingdao University, Qingdao 266071, P.R. China

**Keywords:** Materials in biotechnology, Materials science, Electrochemistry, Bio-electrochemistry

## Abstract

A microbial electrochemical system could potentially be applied as a biosynthesis platform by extracting wastewater energy while converting it to value-added chemicals. However, the unfavorable thermodynamics and sluggish kinetics of *in vivo* whole-cell cathodic catalysis largely limit product diversity and value. Herein, we convert the *in vivo* cathodic reaction to *in vitro* enzymatic catalysis and develop a microbe-enzyme hybrid bioelectrochemical system (BES), where microbes release the electricity from wastewater (anode) to power enzymatic catalysis (cathode). Three representative examples for the synthesis of pharmaceutically relevant compounds, including halofunctionalized oleic acid based on a cascade reaction, (4-chlorophenyl)-(pyridin-2-yl)-methanol based on electrochemical cofactor regeneration, and l-3,4-dihydroxyphenylalanine based on electrochemical reduction, were demonstrated. According to the techno-economic analysis, this system could deliver high system profit, opening an avenue to a potentially viable wastewater-to-profit process while shedding scientific light on hybrid BES mechanisms toward a sustainable reuse of wastewater.

## Highlights

• A microbe-enzyme hybrid bioelectrochemical system was developed
• Wastewater-powered production of high-value chemicals was achieved with the hybrid BES
• High system profit for wastewater-to-chemical production was accomplished
• This hybrid BES is promising for techno-economic feasible wastewater-to-profit process

## Graphical abstract

**Figure:**
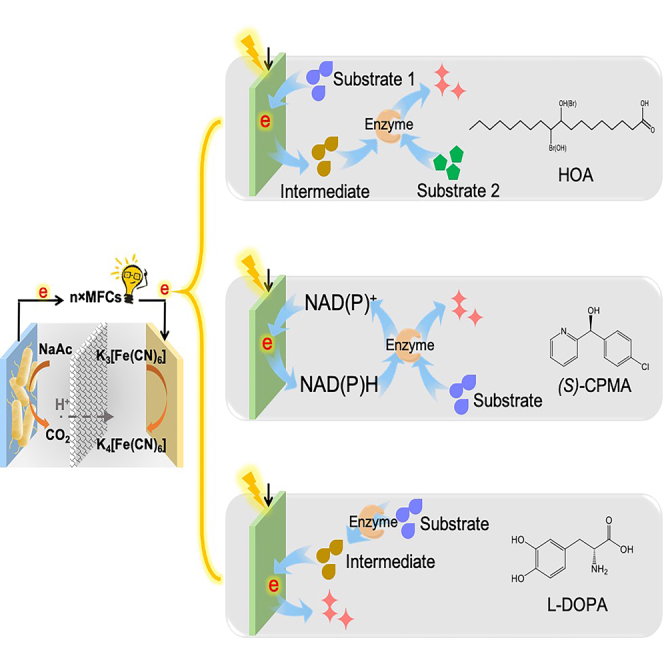

## Introduction

A wastewater-energy-chemical nexus can lead to a sustainable paradigm shift for the wastewater treatment industry, in which wastewater can be employed as a power source for energy production and chemical synthesis (Li et al., 2015; Lu et al., 2018; Mohan et al., 2016; Rabaey and Rozendal, 2010; Zou and He, 2018). When considering the large quantity of global wastewater (1,000 km^3^ yr^−1^) (Heidrich et al., 2011), the large amount of chemical energy that it contains (17.8–28.7 kJ g^−1^ COD) is often overlooked (McCarty et al., 2011). Thus, the wastewater-energy-chemical nexus holds high potential to bring tremendous value to the wastewater industry. Microbial electrochemical systems as a flexible platform technology involve the bioelectricity generation and utilization by electroactive microorganisms through the systems of microbial fuel cells (MFCs), microbial electrolysis cells, microbial electrosynthesis cells, and so on (Logan et al., 2019; Wang and Ren, 2013; Yan et al., 2013; Yong et al., 2014; Zou et al., 2016). Microbial electrosynthesis cells can provide comprehensive solutions for wastewater-energy-chemicals nexus by extracting the wastewater energy and efficiently converting it into various value-added chemicals (Harnisch and Schroder, 2010; Zou and He, 2018). In particular, the integration of microbial electrosynthesis cells with MFCs has accomplished the wastewater-powered production of different commodities, such as biomethane (Ning et al., 2021), acetic acid (Nevin et al., 2010), butanol (Zaybak et al., 2013), and hexanol (Vassilev et al., 2018), thereby opening the window for bringing value to wastewater treatment (Christodoulou et al., 2017; Jiang and Zeng, 2018). A study on microbial electrosynthesis reported that *in situ* electrode potential modulation and control can be achieved through the introduction of additional “‘pin”’ electrodes (Gajda et al., 2021). Recently, the scaling-up feasibility of microbial electrosynthesis has been studied (Zou et al., 2021).

However, the product spectrum of microbial electrosynthesis is restricted to simple commodities with low economic value (Lovley and Nevin, 2013; Wang and Ren, 2013) due to the limitations of extracellular electron transfer (EET) and *in vivo* electron flux control (Yu et al., 2020). First, thermodynamic problems associated with EET largely limit product diversity and productivity (Rabaey and Rozendal, 2010; Shi et al., 2016). The energy gain (driving force for biosynthesis) for microbial electrosynthesis is restricted by the redox potential of the *in vivo* EET machinery (Gildemyn et al., 2017; Harnisch and Schroder, 2010). For example, EET with membrane-embedded cytochrome complexes as an electron carrier cannot thermodynamically drive biosynthesis with a redox potential lower than −0.6 V (versus Ag/AgCl) because the cytochrome complexes only have a redox potential range of −0.6 to 0.3 V (Liu et al., 2014a). In addition, the products were kinetically hampered by a slow EET rate owing to the sluggish electron relay through the cell membrane (Zhang et al., 2018), as well as the limited diffusion process with the use of electron mediators. Furthermore, accurate electron flux control for *in vivo* EET is quite challenging for microbial electrosynthesis. Complicated intracellular metabolic networks make it impossible to seamlessly wire an EET pathway with the target biosynthesis module (Kracke et al., 2018). Therefore, it is unfeasible to simultaneously meet the thermodynamic, kinetic, and control requirements for the production of high-value chemicals based on known *in vivo* microbial electrosynthesis systems.

The bioelectrochemical technology to convert wastewater to value-added products should achieve fast reaction rates, high selectivity of target chemicals, as well as easy separation of the desirable compounds from the catholyte (Gajda et al., 2021). In contrast to *in vivo* microbial electrosynthesis, *in vitro* cell-free enzymatic synthesis provides a simple and flexible alternative way for biosynthesis, with advantages of simple process development, enhanced efficiency, and high flexibility (Al-Shameri et al., 2020; Hodgman and Jewett, 2012; Pardee et al., 2016; Zhu et al., 2014). More importantly, it shows remarkable chemo-/regio-/stereoselectivities, which have great potential for the production of high-value chemicals, including food ingredients, vaccines, and drug precursors (Bornscheuer et al., 2012; Rudroff et al., 2018; Zhang, 2015). In particular, enzymatic electrosynthesis systems powered by electricity have been developed to further improve the flexibility of enzymatic synthesis by using an easily tunable electron supply as the reducing power (Figure S1) (Wu and Zhu, 2018). The use of enzymes as electrocatalysts brings opportunities to meet the thermodynamic and kinetic requirements for the synthesis of diverse high-value chemicals (Chen et al., 2018, 2019). Furthermore, the *in vitro* system is independent of complicated cell metabolism, which allows for relatively easy fine-tuning of the electron flux (Abdellaoui et al., 2018; Chen et al., 2019; Markle and Lutz, 2008). To date, although microbial battery-powered enzymatic electrosynthesis of hydrogen and formate has been reported, the economic values of products remained low (Dubrawski et al., 2019). As such, it is possible to employ an *in vitro* enzymatic electrosynthesis system as an alternative, which may overcome the above-mentioned problems of microbial electrosynthesis and boost the economic feasibility of the overall process (Rabaey, 2020).

**Figure 1:**
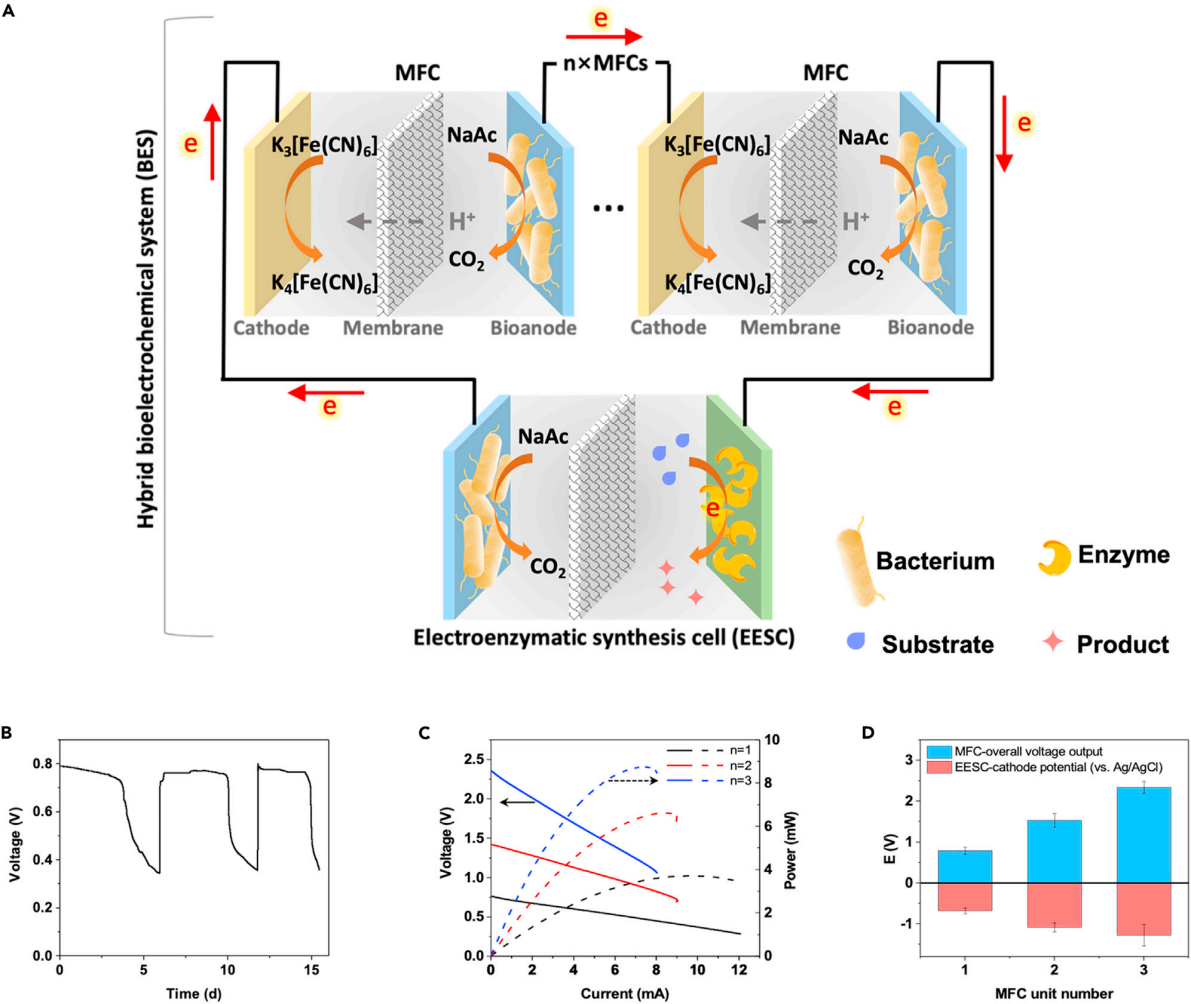
Working principle of the microbe-enzyme hybrid BES for wastewater-powered high-value chemical synthesis (A) Schematic of the electroenzymatic synthesis in a microbe-enzyme hybrid BES powered by wastewater. (B) Voltage of a single MFC running with 2,000 Ω of external resistance during three batch operations. (C) Polarization curves of MFC stacks connected in series (n = 1, 2, and 3). (D) Cathodic potentials of the EESC (versus Ag/AgCl) and output voltages of the MFC stacks connected in series with different unit numbers. Data are represented as means ± standard error of mean.

Herein, we develop an innovative concept and approach to combine the high-value chemical production along with wastewater treatment, demonstrating a microbe-enzyme hybrid bioelectrochemical system (BES) that is established by integrating an MFC stack with an enzymatic electrosynthesis cell (EESC) for wastewater-powered high-value chemical production. The electrons harvested from wastewater by MFCs (anodic reaction) serve as the reducing power for *in vitro* enzymatic synthesis (cathodic reaction) (Figure 1A). In this context, the diluted chemical energy embedded in wastewater can be upgraded into chemical bonds of valuable fine chemicals. The serial modular design enables a simple change in the number of stacked MFCs (n×MFCs) to manipulate the cathodic potential of the EESC, thereby meeting the thermodynamic requirement for different enzymatic reactions (Figure 1A). As a proof of concept, three representative wastewater powering strategies, H_2_O_2_-mediated electron transfer, cofactor-mediated electron transfer, and direct electron injection, are designed to fulfill the requirements of a vast variety of production processes. High-value chemicals, including halofunctionalized oleic acid (HOA, **1b**), (4-chlorophenyl)-(pyridin-2-yl)-methanol (CPMA, **2b**), and l-3,4-dihydroxyphenylalanine (L-DOPA, **3b**), are produced via our hybrid BES. According to the techno-economic analysis proposed in this work with specific assumptions of fixed and variable costs, the hybrid BES could potentially offer tremendously boosted profit through the use of wastewater energy.

## Results and discussion

### Working principles

As shown in Figure 1A, the microbe-enzyme hybrid BES was constructed with a series of stacked MFCs (the number of units (n) is adjustable) and an EESC. The MFC and EESC utilized the same bioanode, which contained an enrichment of electrochemically active bacteria, but different cathodes. The MFC cathode utilized potassium ferricyanide as the electron acceptor, whereas the highly efficient enzymatic cathodes of the EESC were fabricated with specific enzymes for the production of corresponding chemicals. One single MFC produced an output voltage of ∼0.79 V, which was retained for 3–4 days per batch (Figure 1B). An increased number of stacked MFC units tended to produce an increased voltage or power output. When three MFC units were connected, an overall voltage of 2.34 ± 0.14 V and a power of 8.9 ± 0.3 mW were obtained (Figure 1C). In the case of the EESC, a more negative cathodic potential was also obtained with an increased number of MFC units. It can be easily increased from −0.68 ± 0.07 (n_MFC_ = 1) to −1.28 ± 0.18 V (n_MFC_ = 3) with variable MFC stacking (Figure 1D). Moreover, the air-cathode MFC stacking (replacing potassium ferricyanide with an activated carbon paste cathode) showed similar performance, which could achieve a cathodic potential of up to −1.28 ± 0.09 V (n_MFC_ = 4) (Figure S2). These results indicated that a hybrid BES with a tunable cathodic potential for enzymatic transformation was successfully developed.

### Synthesis of hydroxy/halofunctionalized oleic acid via a cascade reaction

**Figure 2:**
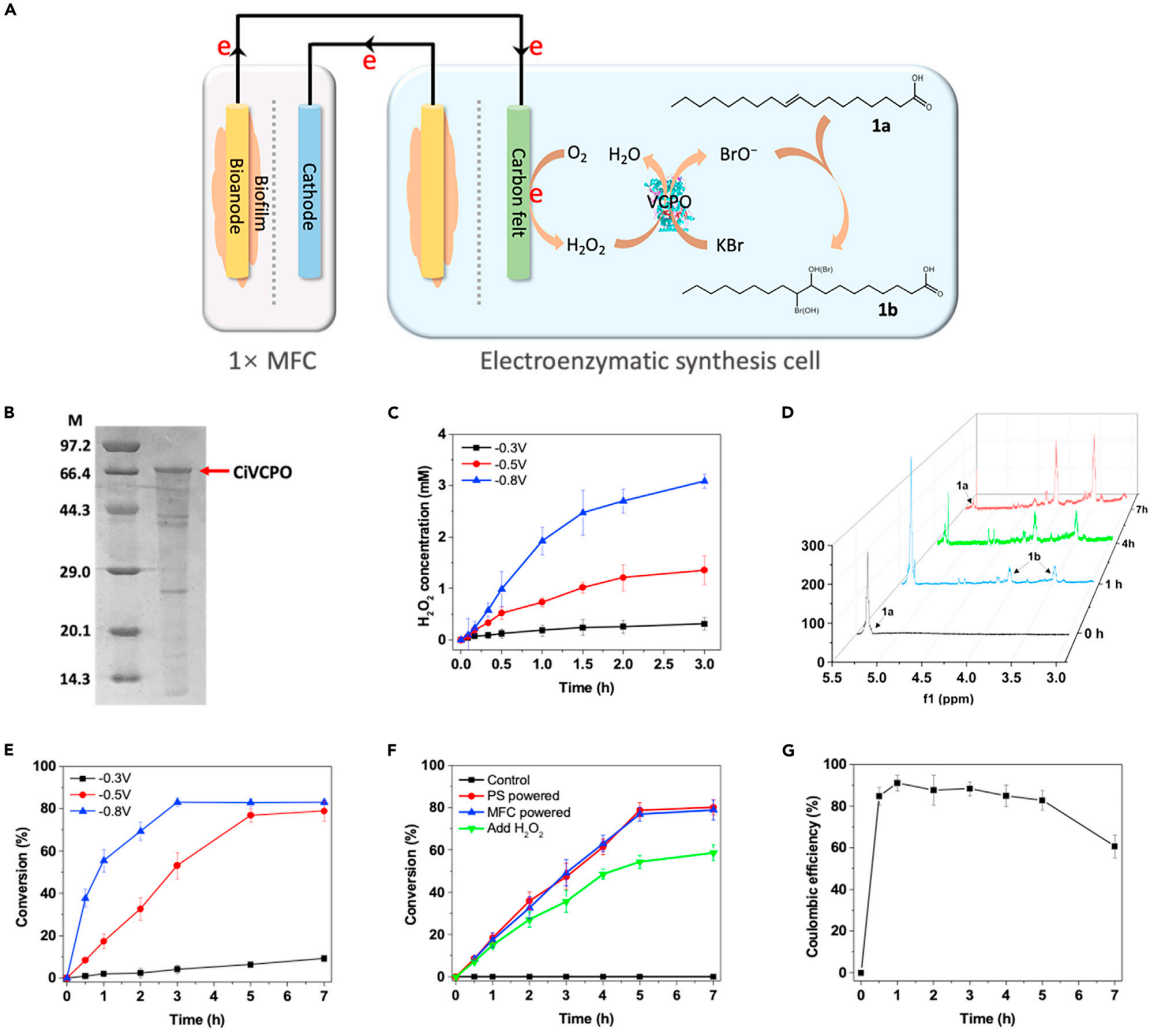
Overview of HOA synthesis based on the hybrid BES (A) Schematic of **1b** production at the cathode of the EESC. (B) Sodium dodecyl sulfate polyacrylamide gel electrophoresis (SDS-PAGE) analysis of CiVCPO after 90 min of heat purification and anion exchange chromatography, which was then used for gel densitometric purity estimation. (C) H_2_O_2_ generation at three potentials, −0.3 (black line), −0.5 (red line), and −0.8 V (blue line), in a three-electrode system with carbon felt as the working electrode and citrate buffer (0.1 M, pH 5.0) as the electrolyte in the presence of O_2_ bubbling. Data are represented as means ± standard error of mean. (D) Nuclear magnetic resonance (NMR) spectra of the **1b** conversion by the hybrid BES after reacting for 0 (black line), 1 (blue line), 4 (green line), and 7 h (red line). A single MFC was used as the power source with a potential of −0.5 V at the EESC cathode and 5 mM **1a** was added. 10-Bromo-9-hydroxyoctadecanoic acid and 9-bromo-10-hydroxyoctadecanoic acid were identified by crude ^1^H NMR spectra. ^1^H NMR (400 MHz, CDCl_3_) δ: 0.85 (3H, m), 1.16–1.87 (26H, m), 2.31–2.36 (2H, m), 3.33–3.35 (1H, m), 4.03–4.05 (1H, m). The results at 3.0–5.5 ppm in the spectra were magnified, and representative peaks in the ^1^H NMR results of **1a** and **1b** were labeled. (E) **1b** yield at potentials of −0.3 (black line), −0.5 (red line), and −0.8 V (blue line) using a three-electrode system in a citrate buffer (0.1 M, pH 5.0) with 40% ethanol as a cosolvent, along with the addition of 200 μM CiVCPO, 5 mM **1a**, and 20 mM KBr. Data are represented as means ± standard error of mean. (F) **1b** conversion rates when using a potentiostat (red line), the MFC (blue line), and the addition of H_2_O_2_ (green line) as the reducing power. The electrolyte was citrate buffer (0.1 M, pH 5.0) with 40% ethanol as a cosolvent; 200 nM CiVCPO, 5 mM **1a**, and 20 mM KBr were added. A control experiment (black line) was conducted under the same condition but without electric power or H_2_O_2_. Data are represented as means ± standard error of mean. (G) Faradaic efficiencies during the hydroxy/halo-functionalization of **1a**. Data are represented as means ± standard error of mean.

Next, the feasibility of MFC-powered chemical synthesis with a hybrid BES was evaluated. Initially, the possibility of activating molecular oxygen for chemical production in a cascade reaction was demonstrated (Figure 2A). A vanadium-dependent chloroperoxidase from *Curvularia inaequalis* (CiVCPO, Figure 2B) (Hasan et al., 2006; Yuan et al., 2020), which can catalyze the hydroxy/halo-functionalization of oleic acid (**1a**) to 10-bromo-9-hydroxyoctadecanoic acid and 9-bromo-10-hydroxyoctadecanoic acid (**1b**, high-value anticancer drugs/antibacterial agents) (Dong et al., 2017), was used as a cathode catalyst of the EESC. CiVCPO is an oxidative enzyme utilizing H_2_O_2_ as a stoichiometric oxidant. Because a simple bulk addition of H_2_O_2_ results in undesirable side reactions and/or the inactivation of enzymes, the electrochemical *in situ* generation of H_2_O_2_ from O_2_ could be used as a viable alternative (Dong et al., 2017). H_2_O_2_ generation was measured at different potentials (−0.3, −0.5, and −0.8 V), which had a positive influence on the H_2_O_2_ production rate (Figure 2C), and faradaic efficiency reached 83.4% (Figure S3). Furthermore, under an optimal enzyme loading of 100 nM (Figure S4), a higher production rate of **1b** was observed at −0.8 V, although the conversion yield turned out to be similar to that of −0.5 V after 5 h (Figures 2D, 2E, and S5). By using the hybrid BES depicted in Figure 2A, which provided a cathode potential of −0.5 V in the EESC, an efficient transformation of **1a** to **1b** was achieved with a high conversion yield of 78.9 ± 4.6% and a conversion rate of 295.9 mg L^−1^ h^−1^; these results are comparable with that of a potentiostat-powered electrochemical process (Figure 2F). The faradaic efficiency of the **1b** conversion (*FE*_*c*_) was estimated to be 91.0 ± 3.7% under optimal conditions (1 h) (Figure 2G and Equation S3), and it decreased after 5 h of reaction because the conversion of **1a** slowed down. On the other hand, the faradaic efficiency for the anode of the MFC (*FE*_*a*_) was below 40% owing to the energy divergence for bacterial growth (Figure S6). In addition, a three-electrode system in one chamber was used as an alternative to the dual-chamber EESC (Figure S7). In this case, a stack of three MFCs was required to achieve a reduction potential of −0.5 V versus Ag/AgCl at the working electrode, and a similar conversion rate of **1b** was observed (Figure S8). These results demonstrated that a hybrid BES could feasibly upgrade the diluted waste energy to high-value chemicals by injecting electrons from the MFC into the cascade reaction of EESC mediated by H_2_O_2_.

### Synthesis of CPMA via electrochemical cofactor regeneration

**Figure 3:**
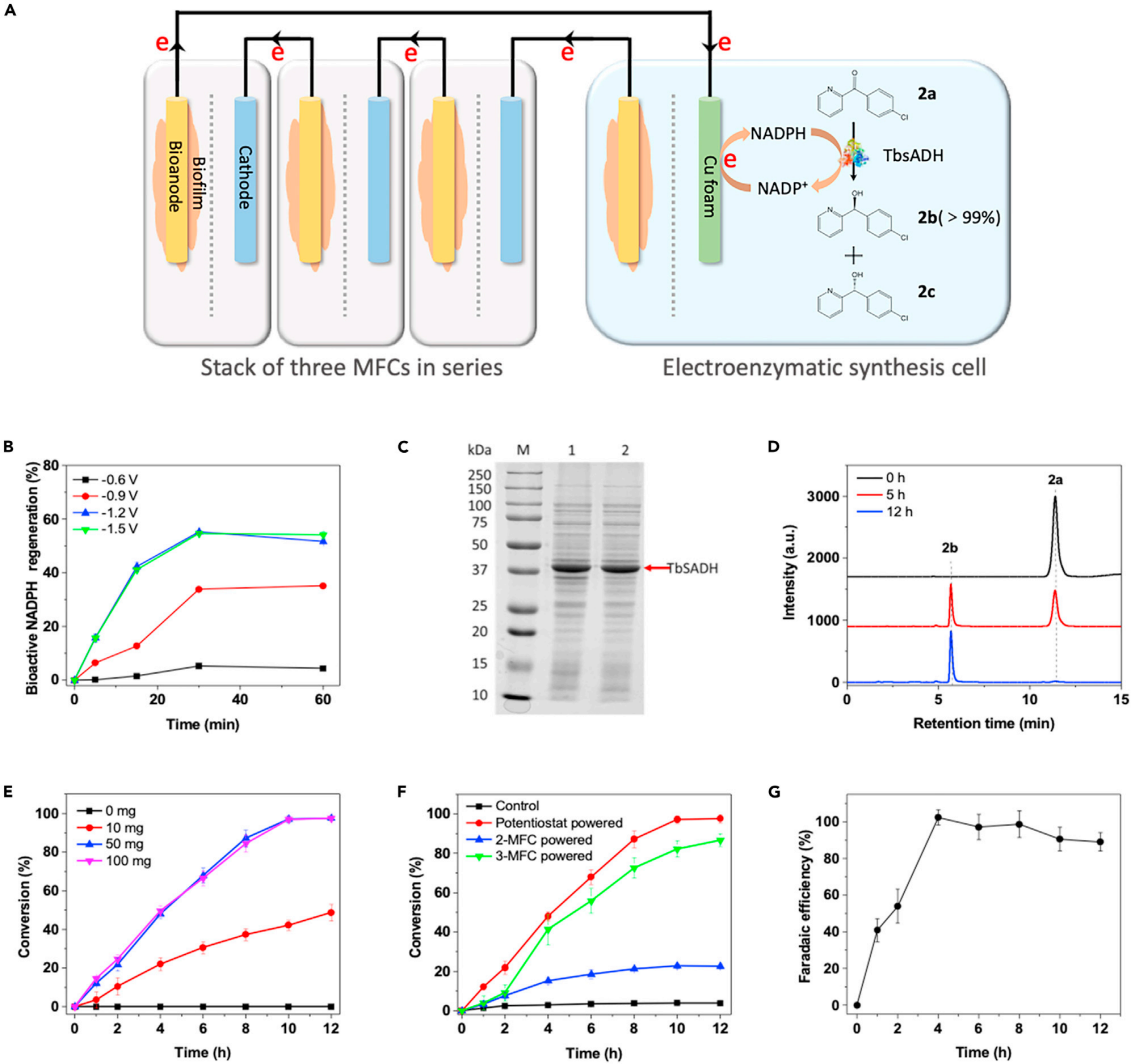
Overview of CPMA synthesis based on the hybrid BES (A) Schematic of **2b** production at the cathode of the EESC. (B) Bioactive NADPH regenerated from the direct electrochemical reduction of 2 mM NADP^+^ via a copper foam electrode in a three-electrode system at four potentials: −0.6 (black line), −1.0 (red line), −1.2 (blue line), and −1.5 V (green line). Data are represented as means ± standard error of mean. (C) SDS-PAGE analysis of TbSADH expression using LB media with 0.1 mM IPTG induction at 20°C. 1, total protein and 2, precipitate of the cell lysis buffer. (D) HPLC profile of **2b** conversion by the hybrid BES after reacting for 0 (black line), 5 (red line), and 12 h (blue line). A stack of three MFCs was used as the power source. Initially, 2 mM NADPH, 20 mM CPMK, and crude TbSADH (∼100 mg protein) were present. Representative peaks of **2a** and **2b** were labeled. (E) **2b** conversion using different enzyme loadings of 0 (black line), 10 (red line), 50 (blue line), and 100 mg (purple line). Initially, 2 mM NADPH, 20 mM CPMK and crude TbSADH (∼100 mg protein) were added to 75 mL of phosphate buffer (pH 7.0, 0.1 M). Data are represented as means ± standard error of mean. (F) **2b** conversion using a potentiostat or the MFC stack (n = 2 and 3), respectively. Initially, 2 mM NADPH, 20 mM CPMK, and crude TbSADH (∼100 mg protein) were present. A control experiment (black line) was conducted under the same conditions but without electric power. Data are represented as means ± standard error of mean. (G) Faradaic efficiencies of the EESC during **2b** synthesis. Data are represented as means ± standard error of mean.

In addition, the donation of electrons with cofactors (e.g., NAD(P)H) is another commonly used process for chemical synthesis (Li et al., 2020; Liu et al., 2019). Thus, *in situ* electrochemical regeneration of cofactors has been increasingly studied in the fine chemical or pharmaceutical industry owing to the convenient control of the reaction process, decreased cost, and elimination of chemical reductants (Ali et al., 2012; Barin et al., 2017). To explore the versatility of the hybrid BES, cofactor regeneration-involved high-value chemical production was investigated (Figure 3A). First, to mimic *in vivo* electron transfer via membrane-embedded cytochrome complexes with a redox potential ranging from ∼−0.6 to 0.3 V (Liu et al., 2014a), a Cu foam electrode was applied for catalytic NADP^+^ reduction at a potential of −0.6 V, in which bioactive NADPH was rarely detected, while the decreased potential (−0.9 to −1.5 V) dramatically elevated the regeneration rate of bioactive NADPH (Figures S9 and 3B). This result is consistent with a previous report that whole-cell MES may not have a sufficiently negative redox potential for electrochemical NAD(H/^+^) regeneration (Li et al., 2018). Thereafter, as an example, an NADPH-dependent alcohol dehydrogenase from the *Thermoanaerobacter brockii* (TbSADH) mutant (Figure 3C) was employed on the cathode of the EESC for the synthesis of (*S*)-(4-chlorophenyl)-(pyridin-2-yl)methanol ((*S*)-CPMA, **2b**) from 4-(chlorophenyl) (pyridin-2-yl) methanone (CPMK, **2a**), which is a crucial chiral synthon of the new antiallergic drugs bepotastine and (*S*)-carbinoxamine (Figure 3A) (Liu et al., 2019). Considering that the reduction of NAD(P)^+^ required a high overpotential, a stack of two or three MFCs had to be used. Under optimal enzyme loading (Figures 3D, 3E, and S10), the conversion of **2b** in the hybrid BES powered by a stack of three MFCs reached as high as 86.8 ± 3.3% after 12 h (which was comparable with that achieved by a potentiostat), whereas hybrid BES with a stack of two MFCs was unable to generate enough cathodic potential to drive this reaction (Figure 3F). The highest *FE*_*c*_ of 102.4 ± 4.1% for **2b** production was obtained after 4 h (Figure 3G). *FE*_*c*_ increased within the first 4 h and decreased afterward because there was very little remaining **2a** that was available. Similar to the example above, the *FE*_*a*_ in this case was below 30% (Figure S11). These results suggested that the hybrid BES could efficiently overcome the thermodynamic limitation by simply adjusting the number of stacked MFCs, demonstrating its great flexibility to upgrade wastewater energy to versatile pharmaceuticals in distinct sophisticated catalytic processes.

### Synthesis of DOPA via enzymatic oxidation coupled with electrochemical reduction

**Figure 4:**
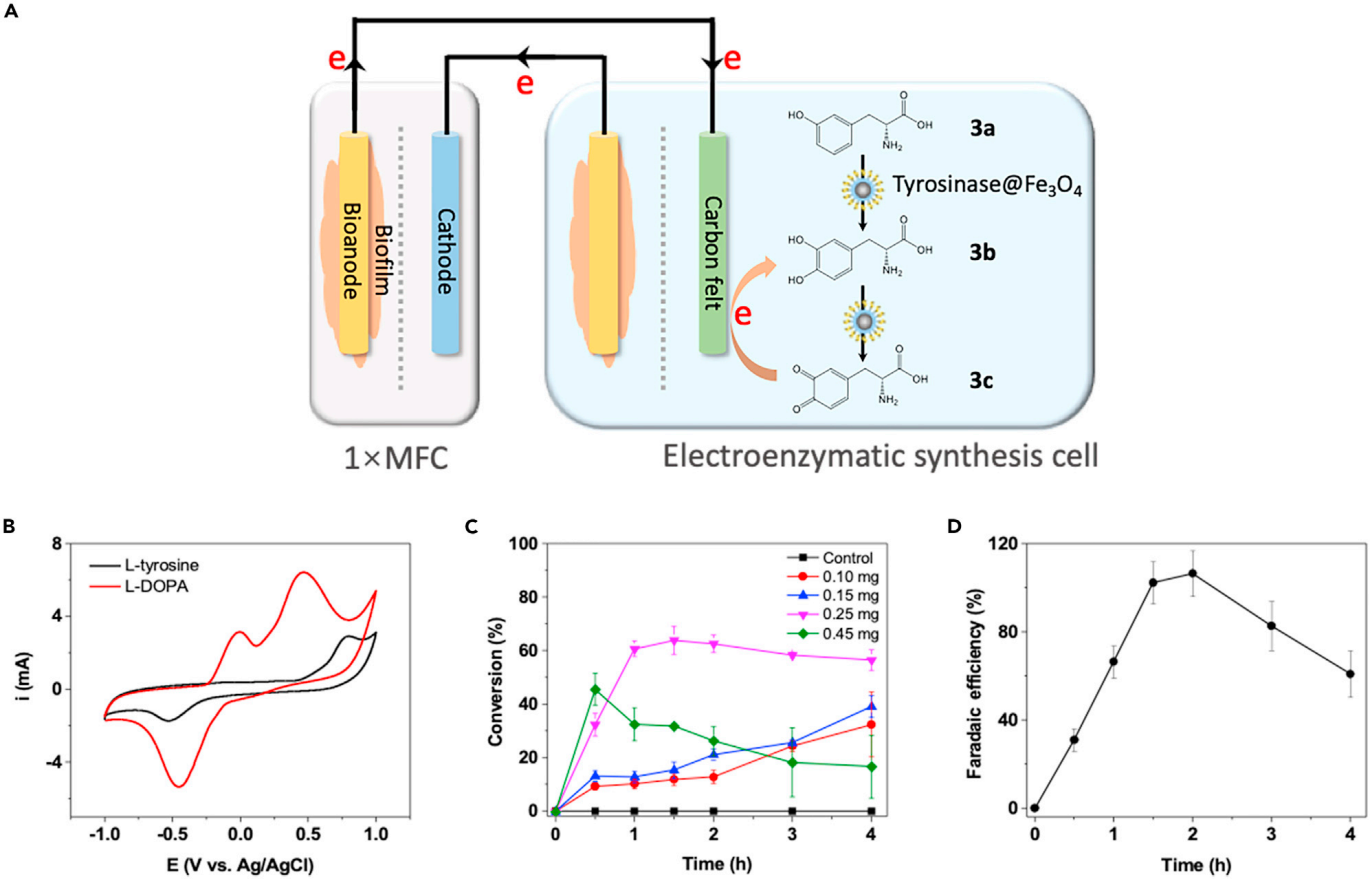
Overview of L-DOPA synthesis based on the hybrid BES (A) Schematic of the electroenzymatic synthesis of **3b** at the cathode of the EESC. (B) Cyclic voltammograms of **3a** and **3b** in a phosphate buffer (0.1 M, pH 7.0) with 50 U of free tyrosinase. Black line, 1 mM **3a**; red line, 1 mM **3b**. Working electrode: carbon felt (1 × 1 cm^2^), reference electrode: Ag/AgCl, counter electrode: Pt sheet (1 × 1 cm^2^), scan rate: 50 mV s^−1^. (C) **3b** synthesis using immobilized tyrosinase@Fe_3_O_4_ in the hybrid BES. Different tyrosinase loadings (0, 0.10, 0.15, 0.25, and 0.45 mg) were used, and 1 mM L-tyrosine was added to 75 mL of phosphate buffer (0.1 M, pH 7.0). Data are represented as means ± standard error of mean. (D) Faradaic efficiencies during the enzymatic electrosynthesis of **3b**. Data are represented as means ± standard error of mean.

In parallel, direct electrochemical transformation-assisted enzymatic synthesis is another widely used process for chemical synthesis. For example, L-DOPA (**3b**), a precursor of dopamine used in the treatment of Parkinson’s disease (Nagatsu and Sawada, 2009), can be synthesized by the enzymatic oxidation of L-tyrosine (**3a**) and a subsequent reduction of the intermediate (Figure 4A). In this context, a hybrid BES was designed with commercial tyrosinases immobilized on the cathode of the EESC (Figure S12), in which a potential of −0.5 V was provided by a single MFC (Figure 4B). The enzyme loading was optimized based on the performance of **3b** production, as the increased loading of tyrosinase resulted in the irreversible conversion of dopaquinone to precipitated leukodopachrome and melanin (Algieri et al., 2012). When 0.25 mg of tyrosinase was immobilized, a maximum conversion of 63.8% was detected at 1.5 h (Figure 4C). An *FE*_*c*_ of 102.2 ± 9.4% for **3b** synthesis at 1.5 h was obtained (Figure 4D). When **3a** was consumed, the enzymatic reaction of **3b** to dopaquinone and the electroreduction of dopaquinone reached equilibrium. Over time, a later decrease in *FE*_*c*_ was observed. Although the *FE*_*a*_ was less than 10% owing to the low current in the cells (Figure S13), these results further provided an alternative using the hybrid BES as a direct reducing power for valuable chemical synthesis.

According to the proof-of-concept demonstration, the thermodynamic and kinetic barriers for the EET and the difficulties in electron flux control can be substantially overcome by the hybrid BES, resulting in a broader spectrum of valuable products than that from the microbial electrosynthesis. High faradaic efficiencies for the electroenzymatic synthesis (*FE*_*c*_) in all three examples indicate the high selectivity of the hybrid BES, which is superior to the current microbial electrosynthesis systems. Moreover, the MFC stack alone could produce a maximum current output of 6–12 mA (n = 1, 2, 3), whereas the highest current output recorded for the hybrid BES is only 5 mA (Figures S6A, S11A, and S13A). The reduced current output in the hybrid BES suggests that the cathode reaction might be kinetically restricted, verifying that such a hybridized system should be powerful enough to drive cathodic high-value chemical synthesis with wastewater energy. Hence, the enhanced electroenzymatic reaction would further improve the overall performance of the hybrid BES. Since the purpose of the study was a proof of concept to construct a system by coupling MFCs to an EESC without supplemental voltage, the reaction conditions for the EESC were not further optimized, as the conversion yields of energy and mass were already high enough. Considering that the context of wastewater treatment is not compatible with the context of pharmaceutical production, which occurs in extremely controlled conditions with quality assurance, matrix effects of wastewater may affect the enzymatic cathode. However, this could be easily solved with chemical processes or/and engineering technology. This route has been demonstrated in Singapore, in which dirty sewage could easily be recycled into drinking water. Other recently reported strategies include the physical separation of these two systems or the use of microbial batteries (Dubrawski et al., 2019). In this study, ion chromatography and inductively coupled plasma mass spectrometry were employed for the detection of anodic representative anions (PO_4_^3−^, NO_3_^-^, CH_3_COO^−^) and cations (Fe^2+^/Fe^3+^, Cu^2+^, Zn^2+^, Pb^2+^), respectively, in the enzymatic cathode by a world-famous pharmaceutical company, WuXi AppTec (Table S1). All ion concentrations were below or approaching the limit of quantitation/limit of detection in the samples from a dual-chamber EESC cathode, demonstrating that high-value chemical production at the cathode would not be affected by wastewater at the anode since there was a proton exchange membrane between the anodic and cathodic chambers.

### Techno-economic analysis

To further justify the feasibility of this proposed hybrid BES that introduced an EESC into the microbial electrochemical system, a techno-economic model was developed (details in the STAR Methods and Table S2) (Christodoulou et al., 2017; Christodoulou and Velasquez-Orta, 2016; Claassens et al., 2019; Rieth and Nocera, 2020). It had to be noted that this model was set to describe the scaled-up production using the hybrid BES in the future and therefore contained many conjectural assumptions. In brief, the overall cost of the hybrid BES was simply divided into fixed costs (including the cost of investment (*C*_*cap*_), maintenance (*C*_*m*_), energy consumption for wastewater feeding (*C*_*pump*_)) and variable costs (including the energy consumption for working potential control (*C*_*pstat*_), cathode catalyst (*C*_*catal*_), feedstock (*C*_*feed*_), and separation (*C*_*sep*_)). Among them, cathode catalyst (enzyme) cost was a crucial and potentially very expensive component for the scale-up system. Based on small-scale preparation of enzymes with low-cost substrates or commercially produced enzymes with high-cost substrates (details in STAR Methods), we estimated a ratio of cathodic catalyst cost to feedstock (η_catal_) ranging from 0.05–0.8. In addition, as we intended to synthesize crude products with high value rather than chemicals of pharmaceutical grade, the cost of pumps and product separation unit with a ratio of 1:1 to the reactor cost was presumptively adopted. To provide a thorough understanding of the cost variation among different hybrid BES systems, the cost was normalized to the charge delivered in the external circuit (kAh).

Our economic model revealed a rather high fixed cost (5.2–25.9 $ kAh^−1^, corresponding to an MFC anode current density of 63.6–6.4 A m^−3^) (Table S3; Figure S14). Moreover, the analysis of the fixed cost variation suggested that it was mainly restricted by the current density and not sensitive to the anodic faradaic efficiency of the MFC. Therefore, the relatively low *FE*_*a*_ shown in the above examples was inconsequential. Instead, a current density higher than 1,000 A m^−3^ was required to lower the fixed cost to the content that could be viable for low-value added chemical commodity production (0.2 $ kAh^−1^) (Figure S15). However, this requirement was difficult to achieve with current MFC technologies (He, 2017; Logan et al., 2015). Therefore, efforts should be made to boost the value of products synthesized at the cathode of the EESC.

**Figure 5:**
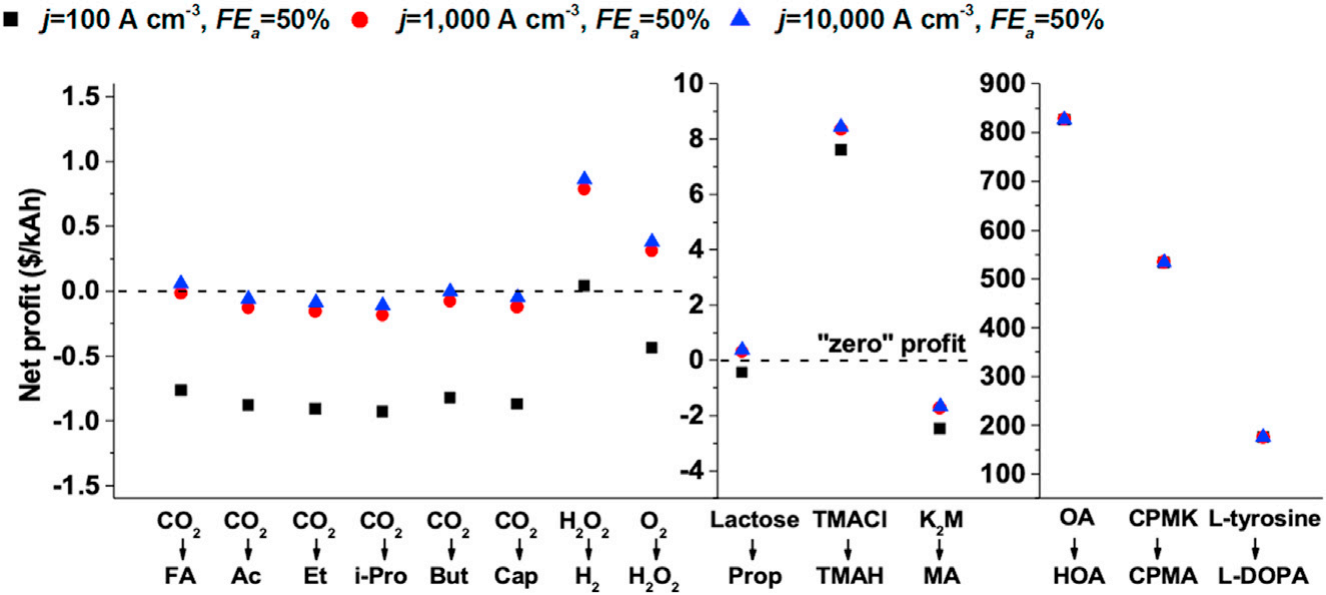
Techno-economic analysis The net profit for the bioelectrochemical refinery of wastewater into typical chemicals is derived from Figures S14 and S15. Current densities of 100, 1,000, and 10,000 A m^−3^ were selected, which represented the current performance at the pilot scale, laboratory scale, and desired target. The “zero” profit indicated the balance of cost and profit, which meant that the net-add value (*V*_*pro*_ - *Y*) only covered the basic cost (*X*). All the costs and profits were calculated based on the electrons transferred across the external circuit in the volumetric wastewater ($ kAh^−1^). FA (formic acid), Ac (acetic acid), Et (ethanol), i-Pro (isopropanol), But (butyrate), Cap (caproate), Prop (propionate), TMAH (tetramethylammonium hydroxide), TMACl (tetramethylammonium chloride), MA (malic acid), K_2_M (potassium malate), HOA (hydroxy/halofunctionalized oleic acid), OA (oleic acid), CPMA ((4-chlorophenyl)-(pyridin-2-yl)methanol), CPMK (ketone 4-chlorophenyl) (pyridin-2-yl)methanone), L-tyrosine, and L-DOPA (l-3,4-dihydroxyphenylalanine) were the analyzed products.

Furthermore, we also applied our economic model to evaluate the cost and benefit of the products from the reported microbial electrosynthesis systems (Figure 5 and the parameters in Table S4), such as acetic acid and ethanol, which still had low economic values and were not sufficient to compensate for the cost of electrical energy and other expenditures (Christodoulou et al., 2017). Although a few moderate-value products, such as tetramethylammonium hydroxide (TMAH), showed positive theoretical benefit, these *in vivo* systems highly relied on microbial metabolism (Jiang and Zeng, 2018), which to a great extent restricted the product spectrum and production efficiency. When the enzymatic reaction was coupled to produce valuable chemicals by the hybrid BES here, high economic profit could be expected of the wastewater-energy-chemical nexus (Figure 5).

Compared with different biosynthesis processes for these three pharmaceutically relevant products, high conversion and productivity were also obtained when using the constructed hybrid BES (Table S5). Previously, similar MFC stacks connected in series or parallel were coupled with an electroenzymatic synthesis to produce formate (Dubrawski et al., 2019; Lijuan Zhang, 2017) or hydrogen (Dubrawski et al., 2019), and few attempts were actually made to produce high-value chemicals. Although our recent work reported a self-powered electroenzymatic synthesis system for **3b** production using enzymatic fuel cells as the power source, enabling the extraction of electrons from sugars, this system was still not economically viable since the sugars were valuable resources in the environment, and the enzymatic reaction for generating electricity also suffered from high cost (Wu and Zhu, 2018). To the best of our knowledge, the additional value obtained in this work to date was estimated as the highest among the numerous wastewater bioelectrochemical conversion systems, with a presumable increase of approximately 100 times compared with that of a conventional microbial electrosynthesis cell design (Figure 5). Moreover, this microbe-enzyme hybrid BES could also be applied for other reactions associated with electron transfer by simply replacing enzymes and constructing the corresponding cathode.

## Conclusions

In summary, we have successfully constructed a microbe-enzyme hybrid BES system by integrating wastewater-fueled MFCs with an *in vitro* cell-free EESC. With the *in vitro* transmembrane EET-free design, electrons from the MFC were seamlessly wired to the reactant, cofactor, or reaction intermediate, which greatly facilitated electron flux control and overcame the thermodynamic and kinetic limitations of *in vivo* microbial electrosynthesis systems. In light of these advantages, the hybrid BES system could synthesize fine chemicals with high-value and complexity. For example, three typical strategies for high-value chemical (HOA, CPMA, and L-DOPA) biosynthesis with the hybrid BES were demonstrated by powering enzymatic catalysis with direct electron transfer toward enzymes, cofactor regeneration, or the direct reduction of intermediates. A techno-economic analysis indicated that the production of valuable chemicals with the hybrid BES could improve the economic feasibility of wastewater bioelectrochemical refineries. Thus, the results here demonstrate that the hybrid BES may provide a promising platform for enzymatic electrosynthesis of high-value compounds powered by sustainable energy extracted from wastewater treatment.

### Limitations of study

One of the key weaknesses for enzymatic catalysts is their short lifetime. Therefore, strategies are required to be applied including properly immobilizing enzymes onto the electrode to increase the reusability and digging out new enzymes with good thermostability. Moreover, facile and mild preparation methods are needed to avoid possible enzyme deactivation. Although we discussed the possible cost for scaling up the system, the techno-economic analysis, based on several assumptions such as enzyme cost and separation cost, may be different from how the system would run practically. On the other hand, the concern on the matrix effects of wastewater and the incompatibility between wastewater treatment and fine chemical synthesis should be addressed in practical. In future work, more complicated enzymatic synthesis cascades could be achieved with the construction of *in vitro* multienzyme systems in EESCs for the synthesis of more desirable products. Further evaluation on the economy and practicability of this hybrid system should also be performed.

## Supporting citations

The following references appear in the supplemental information: Arends et al., 2017; Batlle-Vilanova et al., 2017; Dong et al., 2015; Fu et al., 2010; Galitsky et al., 2008; Jadhav and Ghangrekar, 2009; Jourdin et al., 2018; Lefebvre et al., 2011; Liu et al., 2014, 2015; Min et al., 2010; Min et al., 2013; Mohan et al., 2009; Ni et al., 2012; Schuppert et al., 1992; Srikanth et al., 2018; Truppo et al., 2007; Xiang et al., 2017; Xu et al., 2017; Zhang et al., 2010; Zhao et al., 2012; Zhen et al., 2017; Zheng et al., 2018.

## STAR★Methods

### Key resources table

**Table:** 

REAGENT or RESOURCE	SOURCE	IDENTIFIER
**Bacterial and virus strains**		

*Escherichia coli* TOP 10	Simgen	8302020
*Escherichia coli* BL21	Solarbio	BL21

**Chemicals, peptides, and recombinant proteins**		

Tyrosinase (E.C. 1.14.18.1) from mushroom	Worthington Biochemical Corp.	LS003789
Fe_3_O_4_-COOH magnetic nanoparticles	Shanghai Aladdin Bio-Chem Technology Co., LTD	M120284
Carbon felt	Fuel Cell Earth	G600
Nafion® 212	DuPont	Nafion 212
Activated Carbon	Xinsen Carbon Co., Ltd	N/A
Nickel foam	Kunshan Guang-jia-yuan New Materials Co., Ltd	N/A

**Recombinant DNA**		

pBADgIIIB-VCPO	Yuan et al. (2020)	GenBank: CAA59686.1
pRSFDuet-1-TbSADH	Liu et al. (2019)	N/A

### Resource availability

#### Lead contact

Further information and requests for resources and reagents should be directed to and will be fulfilled by the lead contact, Professor Zhiguang Zhu (zhu_zg@tib.cas.cn).

#### Materials availability

This study did not generate new reagents.

### Experimental model and subject details

#### Stains for enzyme expression

*E. coli* TOP10 containing pBADgIIIB-VCPO vector was grown in 10 mL LB medium containing 50 mg mL^−1^ ampicillin and cultured overnight at 37°C, 180 rpm. The pre-culture (2 mL) was then transferred into 200 mL TB medium (tryptone 12 g L^−1^, yeast extract 24 g L^−1^ and glycerol 5 g L^−1^ in 89 mM pH 7.5 KPi buffer) supplemented with 50 mg mL^−1^ of ampicillin and grown at 37°C, 180 rpm. L-arabinose (0.02 wt%) was added at an OD_600_ of 0.9 for induction and the culture was incubated for additional 24 h at 25°C, 180 rpm.

*E. coli* BL21 containing pRSFDuet-1-TbSADH cell was grown in LB medium containing 50 μg mL^−1^ kanamycin. The cells were cultured at 37°C with shaking and a final concentration of 0.1 mM IPTG was added to the culture when OD_600_ reached to 0.6–0.8. The cultivation continued for 16 h at 20°C with shaking for protein expression.

### Method details

#### Construction of the MFCs and EESCs

Dual chamber “H” MFC separated by a proton exchange membrane (Nafion® 212, DuPont Co., USA) was used in the present study. Carbon felt electrodes (2 × 3 cm^2^, 5 mm thick) were suspended in the anode and cathode chambers. The EESC consisted of a similar bioanode of the MFC and a modified cathode for electroenzymatic synthesis. The volume for each chamber of the MFC and the EESC was 75 mL. To setup the hybrid BES, the microbial anodes in both MFC and EESC were enriched with electrochemically active microbes by inoculating activated sludge at 30°C. The microbes were fed with simulated wastewater prepared in phosphate buffer (pH 7.0, 50 mM) containing: 1 g L^−1^ CH_3_COONa, 0.5 g L^−1^ NaCl, 1 g L^−1^ NH_4_Cl, 95.2 mg L^−1^ MgCl_2_, 11.1 mg L^−1^ CaCl_2_, Wolfe mineral 10 ml L^−1^, Wolfe vitamin 10 ml L^−1^. Wolfe Mineral (per liter) contained: 2.14 g nitrilotriacetic acid (NTA), 0.1 g MnCl_2_·4H_2_O, 0.3 g FeSO_4_·7H_2_O, 0.17 g CoCl_2_·H_2_O, 0.2 g ZnSO_4_·7H_2_O, 0.03 g of CuCl_2_·2H_2_O, 5 mg KAl(SO_4_)_2_·12H_2_O, 5 mg of H_3_BO_3_, 0.09 g Na_2_MoO_4_, 0.11 g NiSO_4_·6H_2_O, and 0.02 g of Na_2_WO_4_·2H_2_O. Wolfe’s Vitamin (per liter) contained: 2.0 mg biotin, 2.0 mg folic acid, 10.0 mg pyridoxine HCl, 5.0 mg riboflavin, 5.0 mg thiamine, 5.0 mg nicotinic acid, 5.0 mg pantothenic acid, 0.1 mg cyanocobalamin, 5.0 mg p-aminobenzoic acid, and 5.0 mg thioctic acid. Phosphate buffer (pH 7.0, 50 mM) containing 50 mM potassium ferricyanide was used in the cathode. An external resistance of 2000 Ω was applied and stable electricity output could be obtained after a one-month startup operation.

#### Construction of air-cathode MFC

The air cathode consisted of gas diffusion layer and catalytic layer was fabricated according to previous report (Dong et al., 2012) with minor modification. Firstly, 4 g carbon black was dispersed into 100 mL pore-former ethanol, ultrasonicated in a flask for 30 min at 30°C, followed by slow addition of 10 g PTFE suspension (60 wt%) with ultrasonic agitation for another 30 min. Then, the blend was heated in water bath at 80°C to remove excess ethanol and a dough-like paste was obtained. The black paste was rolled into a ∼0.35 mm-thick sheet and then transferred onto a nickel foam plate (4 × 4 cm^2^). The sheet was then sintered at 340°C for 25 min to melt PTFE in order to obtain the gas diffusion layer. For catalytical layer fabrication, a blend of activated carbon and PTFE (mass ratio = 6:1) was also rolled into a ∼0.35 mm-thick sheet, coated onto the surface of the gas diffusion layer, and sintered at 340°C for 25 min to obtain the integrated air cathode. To construct an air-cathode MFC, a single-chamber cell was used, in which the bioanode and the air-cathode were equipped at opposite ends without the separation of membrane.

#### Electrochemical characterization of MFC

Electrochemical tests were performed by using a CHI 660E potentiostat (CH Instruments Inc., Shanghai, China). All potentials mentioned in this work were versus Ag/AgCl. Voltage of a single MFC running with a 2000 Ω external resistance during three batch operations was recorded. Open circuit potentials of a single MFC or MFC stacks were measured and linear sweep voltammetry was carried out at a scan rate of 1 mV s^−1^. The power was calculated by the current and potential recorded.

#### Electroenzymatic synthesis in the hybrid BES

For **1b** synthesis, a single MFC was used as the power source, and a CF electrode was used as the cathode of the EESC. Oleic acid at a final concentration of 5 mM was used as the substrate, and 20 mM KBr and 200 nM CiVCPO were added to 75 mL of citrate buffer (pH 5.0, 0.1 M) at the cathode of the EESC. The temperature was controlled at 30°C using a thermostatic incubator. Regarding **2b** synthesis, an MFC stack (n = 2 or 3) connected in series was employed as the power source, and a 2 × 3 cm^2^ Cu foam was used as the cathode of the EESC. Final concentrations of 20 mM CPMK, 2 mM NADPH and crude TbSADH (∼100 mg protein) in 75 mL of phosphate buffer (pH 7.0, 0.1 M) were added to the cathode of the EESC at 30°C. Regarding **3b** synthesis, a single MFC serving as the power source was connected in series with an adjustable resistor and an EESC. A 2 × 3 cm^2^ CF was used as the cathode of the EESC, in which 1 mM L-tyrosine was used as the substrate in 75 mL of phosphate buffer (pH 7.0, 0.1 M). Immobilized tyrosinase@Fe_3_O_4_ or free tyrosinase was added. The potential of the cathode was precisely controlled at −0.5 V using a Ag/AgCl electrode by regulating the external resistance.

#### Electrochemical measurement

Cyclic voltammetry, optimizations of enzyme loading and the applied potential for the synthesis of the three chemicals were carried out with a CHI 660E electrochemical working station in a three-electrode system. For **1b**, **2b** and **3b** synthesis, CF, Cu foam and CF were used as the working electrode, respectively, with Ag/AgCl as the reference electrode and a Pt sheet as the counter electrode. An anaerobic environment was maintained by bubbling with nitrogen. When using a CHI 660E potentiostat as the power source of the EESC, constant potentials of −0.5 V, −1.2 V and −0.5 V were held for the synthesis of **1b**, **2b** and **3b**, respectively.

#### H_2_O_2_ generation

Potentiostatic method was applied on a CHI 660E potentiostat in a three-electrode system with a CF (1 × 1 cm^−2^) as the working electrode, Ag/AgCl as the reference electrode and a Pt sheet as the counter electrode. Sodium citrate (pH 5.0, 0.1 M) served as the electrolyte. Saturated oxygen environment was maintained by bubbling oxygen. Constant potentials of −0.8 V, −0.5 V and −0.3 V were held for oxygen reduction, respectively.

#### H_2_O_2_ quantitative analysis

H_2_O_2_ concentration was detected by using a glucose determination kit (Beijing Applygen Gene Technology Co. LTD, Beijing, China). In details, R1 and R2 work solutions were pre-mixed with a ratio of 4:1, then samples (20 μL) were added into the mixture (780 μL) for chromogenic reaction at 37°C, 20 min and the absorbance at 550 nm was measured. H_2_O_2_ standard curve drawn from known concentrations was linear with a regression coefficient (R^2^) of 0.9996.

#### CiVCPO expression and purification

*E. coli* TOP10 containing pBADgIIIB VCPO vector was cultured in TB medium for VCPO expression (details in Experimental model and subject details). The bacterial pellets were harvested by centrifugation at 6000 rpm, 4°C for 10 min and re-suspended in 50 mM Tris/H_2_SO_4_ buffer (pH 8.1). Phenylmethylsulfonyl fluoride (0.1 mM, 100 mM stock in isopropanol) was added to the re-suspended cells, which were ruptured by sonication on ice. The samples were then centrifuged (8000 rpm, 20 min) and the supernatant was incubated at 70°C for 1.5 h. After centrifugation (8000 rpm, 10 min), the clarified protein was further purified with a Q Sepharose FF column. After washing with 2 column volumes of Tris/H_2_SO_4_ (50 mM, pH 8.1) and 2 column volumes of NaCl (0.1 M in 50 mm Tris/H_2_SO_4_, pH 8.1), the enzyme was loaded at 7.5 mL min^−1^ and thereafter eluted with NaCl (0.6 M in 50 mM Tris/H_2_SO_4_, pH 8.1). Fractions containing CiVCPO were concentrated (Amicon 10 kDa cut-off membrane), and desalted by using HiTrap desalting or PD10 columns (GE Healthcare) and Tris/H_2_SO_4_ (50 mM, pH 8.1) containing 100 mM orthovanadate.

#### NMR analysis for halofunctionalized oleic acid

To quantitatively determine the turnover rate of halofunctionalized oleic acid, samples from reaction solutions (10 mL) were extracted with an equal volume of dichloromethane. The extracts were pooled and evaporated to dryness at room temperature. The residue was dissolved in chloroform-d (CDCl_3_) and subjected to a NMR spectrometer (Quantum-I, 400MHz, Q. One Instruments Ltd., Wuhan, China).10-bromo-9-hydroxyoctadecanoic acid and 9-bromo-10-hydroxyoctadecanoic acid were identified by crude 1H NMR spectra.

#### TbSADH mutant expression

The TbSADH mutant used in the present study was obtained in a previous work (Liu et al., 2019). *E. coli* cell growth and TbSADH protein expression were performed in LB medium (details in EXPERIMENTAL MODEL AND SUBJECT DETAILS). The cell pellets were harvested by centrifugation (6000 rpm, 10 min) at 4°C and washed with PBS (50 mM, pH 7.4). Then, the pellets were re-suspended in PBS and disrupted by a high-pressure homogenizer. The crude lysate was centrifuged for 8000 rpm, 15 min at 4°C and the obtained supernatant was used for reaction.

#### NADPH regeneration

The oxidoreduction of NADP^+^ was measured by cyclic voltammetry in a three-electrode system with glassy carbon electrode (GCE, Φ3 mm), Ag/AgCl and Pt sheet (1 × 1 cm^2^) served as working electrode, reference electrode and counter electrode in 5 mL phosphate buffer (pH 7.0, 0.1 M). The solution contained 4 mM NADP^+^ and was bubbled with nitrogen during the electrochemical experiment to maintain an oxygen-free environment. The scan rate was 10 mV s^−1^.

For cofactor regeneration, copper foam (2 × 3 cm^2^), Ag/AgCl and Pt sheet (1 × 1 cm^2^) were used as working electrode, reference electrode and counter electrode in a 75 mL three-electrode system, respectively. Phosphate buffer (pH 7.0, 0.1 M) served as the electrolyte and 2 mM NADP^+^ was added. A constant potential of −1.2 V vs. Ag/AgCl was held when using a potentiostat (PS) as the power source. For the three-MFC stack powered system, copper foam was used as the cathode. The solution was bubbled with nitrogen during the electrochemical experiment to maintain an oxygen-free environment.

Biological active NADPH was quantified using an enzyme reaction:

$$\text{NADPH}+{\text{DCIP}}_{\left(\text{ox}\right)}\overset{\text{DI}}{\to }{\text{NADP}}^{+}+{\text{DCIP}}_{\left(\text{red}\right)}$$

Specifically, samples containing bioactive NADPH regenerated by electrochemistry were taken out from electrochemical cells and reacted with 100 μM oxidized dichlorodiisopropylether (DCIP) catalyzed by 160 μg mL^−1^ diaphorase (DI) in a phosphate buffer (0.05 mM, pH 7.0) at room temperature. The blue color of oxidized DCIP was paled after being reduced by NADPH and the absorbance at 600 nm was measured. NADPH standard curve drawn from known concentrations was linear with a regression coefficient (R^2^) of 0.9926.

#### HPLC analysis for CPMA

To quantitatively determine the turnover rate of CPMA, representative samples from reaction solutions (200 μL) were extracted thrice with an equal volume of ethyl acetate. The extracts were pooled and evaporated to dryness at room temperature. The residue was dissolved in 200 μL of isopropanol and subjected to HPLC analysis via an Agilent ZORBAX SB-C18 analytical column (5 μm, 4.6 × 250 mm) and a photodiode array detector. A mixture of acetonitrile and water with a volume ratio of 50: 50 was used as the mobile phase at a flow rate of 1 mL min^−1^. UV absorption at 220 nm was used and the temperature was controlled by a column oven at 30°C. The retention time of CPMK and CPMA was 11.3 min and 5.8 min, respectively.

#### Immobilization of tyrosinase

Tyrosinase was immobilized on Fe_3_O_4_-COOH magnetic nanoparticles via amide bond. The Fe_3_O_4_-COOH magnetic nanoparticles were activated using EDC/NHS for 12 h at room temperature. 800 μL free tyrosinase (0.63 g L^−1^) was then mixed with 200 μL activated Fe_3_O_4_-COOH solution (5 g L^−1^). The mixture was shaken using a vortex mixer for 24 h under room temperature. Tyrosinase@Fe_3_O_4_ was collected by centrifugation (12000 rpm, 2 min) and washed by PBS (pH 7.0, 0.1 M) to remove non-immobilized enzymes. Tyrosinase@Fe_3_O_4_ were re-suspended in 1 mL PBS (pH 7.0, 0.1 M). Bradford assay was employed to detect enzyme concentration in the solution during immobilization and to calculate immobilized enzyme amount by subtraction method. The immobilized tyrosinase was determined to be 0.45 g L^−1^ in the tyrosinase@Fe_3_O_4_ solution.

#### Cyclic voltammetry of L-tyrosine and L-DOPA

Cyclic voltammetry was carried out on a CHI 660E potentiostat in a three-electrode system with carbon felt (1 × 1 cm^2^) as the working electrode, Ag/AgCl as the reference electrode and a Pt sheet as the counter electrode. Phosphate buffer (pH 7.0, 0.1 M) served as the electrolyte and 50 U free tyrosinase was added. Scan rate was 50 mV s^−1^.

#### L-DOPA quantitative analysis

L-DOPA concentration was detected using the modified Arnow method (Ates et al., 2007; Tuncagil et al., 2009). In short, 0.5 mL sample taken from the reactor was mixed with an equal volume of a solution containing 15% sodium nitrite and 15% sodium molybdate, followed by centrifugation (12,000 rpm, 2 min). Sodium nitrite can react with L-DOPA to generate a yellow-colored complex and sodium molybdate was used to prevent the complex decomposition. The absorbance at 460 nm was measured precisely after 1 h. L-DOPA standard curve drawn from known concentrations was linear with a regression coefficient (R^2^) of 0.982.

#### Analysis for sodium acetate

The sodium acetate was analyzed by high performance liquid chromatography (HPLC) (Shimadzu LC-20A; Shimadzu Corporation; Tokyo, Japan) via a Bio-Rad Aminex HPX-87H Column (9 μm, 300 × 7.8 mm) and a refractive index detector (RID-20A) with a mobile phase of H_2_SO_4_ at a flow rate of 0.6 mL min^−1^. The column temperature was controlled at 60°C. The retention time of sodium acetate were 14.8 min.

#### Detection of anions and cations

Representative sewage anions and cations including 5 mg L^−1^ zinc acetate, 10 mg L^−1^ FeCl_3_, 45 mg L^−1^ NH_4_NO_3_, 1 mg L^−1^ PbSO_4_, and 2 mg L^−1^ CuSO_4_ were added into the simulated wastewater of MFC and EESC anodes. The prepared MFC was used as the powered source for HOA synthesis in the prepared EESC or in a single-cell three-electrode system. Simultaneously, the above mentioned EESC with open circuit was also conducted. Ion chromatography (LC) and inductively coupled plasma mass spectrometry (LCPMS) were employed respectively for the detection of anodic representable anions (PO_4_^3−^, NO_3_^−^, CH_3_COO^−^) and cations (Fe^2+^/Fe^3+^, Cu^2+^, Zn^2+^, Pb^2+^) in the enzymatic cathode of EESC by WuXi AppTec (Shanghai, China).

#### Calculation for energy efficiency

The faradaic efficiencies of the hybrid BES were divided into the anodic faradaic efficiency of the MFC stacks (*FE*_*a*_) and the cathodic faradaic efficiency (*FE*_*c*_) of the EESC. The values were dependent on the current in the circuit, the consumption of acetate and the amount of the product. For well-developed MFCs fed with defined wastewater, stable current output with volumetric current density of *j* (A m^−3^) can be achieved, and the totally transferred charge (*Q*) is described as:

(Equation 1) $$Q=j\times V_{a}\times t$$

Where, *V*_*a*_, and *t* refer to the volume of anode and working time, respectively.

For electricity production, the anodic faradaic efficiency of the MFC stacks (*FE*_*a*_) is calculated as follows:

(Equation 2) $$FE_{a}=\frac{Q}{8\times F\times \Delta n\left(NaAc\right)}\times 100\%$$

where 8 represents the number of electrons produced during the oxidation of one molecule of sodium acetate, F represents the Faraday constant (96,485 C mol^−1^), and Δn_(NaAc)_ represents the moles of sodium acetate consumed.

For electroenzymatic synthesis, the cathodic faradaic efficiency (*FE*_*c*_) of the EESC is calculated as follows:

(Equation 3) $$FE_{C}=\frac{n\text{F}\times {\text{n}}_{\left(\text{product}\right)}}{\text{Q}}\times 100\text{\%}$$

where *n* represents the number of electrons transferred during the formation of one molecule of product, F represents the Faraday constant (96,485 C mol^−1^), and n_(product)_ represents the moles of enzymatically electro-synthesized product.

#### The economic model for bioelectrochemical biorefinery of wastewater

An economic model for wastewater biorefinery with bioelectrochemical techniques was developed. The cost of whole process can be divided as fixed cost and variable cost. The former covers the cost for investment, maintenance and the basic energy consumption, which can be viewed as constant for well-developed bioanode and independent of specific cathodic reaction. Whereas, the latter covers the additional cost associated with cathodic reaction. This model intends to reveal how key parameters of bioelectrochemical systems, in particular the current density, influence the overall cost of wastewater biorefinery with bioelectrochemical techniques. Instead, it is not supposed to give a sophisticated techno-economic analysis of producing versatile chemicals.

#### The basic scenario and assumptions

Several techno-economic models of bioelectrochemical synthesis were developed, which mainly focused on the bioelectrochemical synthesis of chemicals from electrochemical CO_2_ reduction (Christodoulou et al., 2017; Christodoulou and Velasquez-Orta, 2016; Claassens et al., 2019). In most of previous work, the synthesis of chemicals in biocathode was coupled with anodic oxygen evolution reaction (OER). As a result, large working voltage (*U*_*cell*_*,* 1–3 V) was required and energy cost was high. However, the high energetic requirement of OER approximately resulted in the over 90% electricity input consumed in the anode. Kenis *et. al*., recently proposed that electro-oxidation of glycerol can be a promising alternative anodic reaction in these bioelectrochemical systems since it reduced the electricity consumption (Verma et al., 2019). Meanwhile, feeding the electroactive biofilm colonized bioanode with wastewater can offer a similar advantage, in which the anodic electron can be harvested at rather low energy cost (Zou and He, 2018). Regardless the distinctive electron transfer pathways between intracellular metabolism of versatile electroactive bacteria and electrode surface, redox cofactors such as NADH were generally adopted as the electron intermediator in this process (Kracke et al., 2015), meaning that harvested electron possessed mild reducing force (*E*^*ϴ*^ *NADH/NAD*^*+*^ = −0.32 V). As a result, if taking CO_2_ to formic acid (*E*^*ϴ*^ *HCO*_*2*_*H/CO*_*2*_ = −0.61 V, pH = 7) (Benson et al., 2009) as the presumed cathodic reaction, the overall reaction and corresponding standard cell voltage (*U*^*o*^) can be defined:

NADH + CO_2_→ NAD^+^ + HCOO^−^*U*^*o*^ = 0.29 V

This *U*^*o*^ is significantly smaller than that with OER anode (1.12 V) (Claassens et al., 2019). Moreover, for well-developed bioanode, the current output is ideally fitted by Nernst-Monod equation (Torres et al., 2008):

(Equation 4) $$j=j_{max}\times \frac{S}{K_{S}+S}\times \frac{1}{1+\text{exp}\left(-\frac{F}{RT}\eta_{a}\right)}$$

Where, *S* is the concentration of electron donor, *K*_*s*_ is the apparent half-maximum-rate concentration and *η*_*a*_ is the anodic overpotential. The ideal fitting of Nernst term means that the anodic current can reached 98% of the maximum value even a small overpotential of 100 mV is applied. The small *U*^*o*^ and anodic overpotential indicate significantly reduced energy consumption compared with an OER anode (Zou and He, 2018). On the other hand, the current density of wastewater fed bioanode is much smaller than water splitting (∼10^0^ *vs.* 10^3^ A m^−2^) (Yu et al., 2017), which might limit the product yield rate and increase the investment and maintenance cost of unit product. Therefore, the economic feature of using bioelectrochemical system for wastewater refinery is shaped by the compromise of reduced energy cost but increased investment and maintenance cost.

To develop a detailed economic model, the cost of investment (*C*_*cap*_), maintenance (*C*_*m*_), energy consumption for wastewater feeding and working potential control (*C*_*pump*_ and *C*_*pstat*_), cathode catalyst (*C*_*catal*_), feedstock (*C*_*feed*_) and separation (*C*_*sep*_), as well as the value of product (*V*_*p*_) were considered. *C*_*cap*_, *C*_*m*_ and *C*_*pump*_ can be viewed as the cost for electricity generation in anode and other basic operation, which is almost independent with the type of cathodic reaction. Whereas, *C*_*pstat*_, *C*_*catal*_, *C*_*feed*_ and *C*_*sep*_ cover the cost for specific cathodic reaction. Herein, the sum of *C*_*cap*_, *C*_*m*_ and *C*_*pump*_ can be viewed as fixed and is denoted as term *X*, while the sum of *C*_*pstat*_, *C*_*cap*_, *C*_*m*_ and *C*_*energy*_ is variable with cathodic reaction and are denoted as term *Y*. The details of all involved parameters are listed in Table S1.

#### Estimation of fixed cost X

For well-developed bioanode fed with defined wastewater, the totally transferred charge (*Q*) can be calculated by stable current output with volumetric current density of *j* (A m^−3^) (Equation 1) and anode faradaic efficiency of *FE*_*a*_ can be achieved (Equation 2). Hence, the treated wastewater (*V*) are described as:

(Equation 5) $$V=\frac{Q\times M_{w,\;o_{2}}}{4\times \Delta COD\times F\times FE_{a}}$$

Where, *Mw, O*_*2*_, *ΔCOD* and *F* refer to the molecular weight of oxygen, consumed COD and Faraday constant (96485 C mol^−1^), respectively.

Recently, with the reduced cost for electrode and membrane, it was suggested that the construction of basic bioelectrochemical reactor can be obtained at 1200–1500 $ m^−3^ (Escapa et al., 2012; He et al., 2019). Herein, a value of 1200 $ m^−3^ that excludes the cost for cathodic catalyst of the electrosynthesis cell (varies with cathodic reaction) is proposed. Also, the rest accessory equipment such as pumps and product separation unit were assumed as 1:1 to the cost of reactor. To cover the *C*_*cap*_ over the running time of the reactor, a simple financial model was adopted from the previous work, in which fixed payment at equal intervals was assumed. Here, interest rate of 5% on the capital investment and a loan term of 20 years is presumed to calculate the annual *C*_*cap*_ as a fixed-rate mortgage (Table S2). Also, annual *C*_*m*_ is assumed to be 5% of capital investment. Hence, total *C*_*cap*_ and *C*_*m*_ can be viewed as constant while total *C*_*pump*_ depending on *V* and can be expressed as:

(Equation 6) $$C_{pump}=p_{elec}\times P_{pump}\times V$$

Where, $p_{elec}$ and $P_{pump}\;$refer to the price of electricity and electricity power consumption for pumping volumetric wastewater. Therefore, fixed cost *X* for unit transferred charge can be defined:

(Equation 7) $$X=\frac{C_{cap}+C_{m}}{Q}+\frac{C_{pump}}{Q}=\frac{C_{cap}+C_{m}}{j\times V_{a}\times t}+\frac{p_{elec}\times P_{pump}\times M_{w,\;o_{2}}}{4\times \Delta COD\times F\times FE_{a}}$$

Where, *j* and *FE*_*a*_ are two intrinsic parameters which reflect the current generation capacity of anode. Unsurprisingly, larger *j* and *FE*_*a*_ are advantageous for small *X*, in which the *C*_*cap*_ + *C*_*m*_ and *C*_*pump*_ for unit transferred charge are directly reduced.

To test the sensitivity of term *X*, the influence of the viable *j* (100 A m^−3^ and 10,000 A m^−3^) and *FE*_*a*_ (10% and 80%) on the relative contribution of (*C*_*cap*_ + *C*_*m*_) and *C*_*pump*_ were investigated (Table S3). The simulated results indicate that *C*_*cap*_ and *C*_*m*_ are the main source of X at low current density scenario whereas at high current density such as 10,000 A m^−3^, the cost for wastewater pumping significantly increases, especially when *FE*_*a*_ is small.

#### Estimation of variable cost Y

According to the previous report, the additional energetic cost (*C*_*pstat*_) varies with cathodic reaction, and can be estimated as:

(Equation 8) $$C_{pstat}=p_{elec}\times U_{cell}\times Q\;$$

Where, *U*_*cell*_*×Q* refers to the theoretical energetic consumption to power up electricity to drive specific cathodic reactions. Also, with *U*^*o*^, *U*_*cell*_ and cathodic faradaic efficiency (*FE*_*c*_), energetic efficiency (*EE*), which depicts the ratio of energy gain in the overall reaction to the input bioelectric energy, can be further defined and *C*_*pstat*_ can be also expressed as:

(Equation 9) $$EE=\frac{U^{o}}{U_{cell}}\times FE_{c}$$

(Equation 10) $$C_{pstat}=\frac{p_{elec}\times U^{o}\times FE_{c}\times Q}{EE}\;$$

If only the main raw material is counted, stoichiometric cost for feedstock (*C*_*feed,0*_) can be expressed as:

(Equation 11) $$C_{feed,0}=p_{feed}\times n^{\prime }\times m_{pro}\times M_{w,\;feed}$$

(Equation 12) $$m_{pro}=\frac{Q\times FE_{c}}{n\times F}$$

Where, $p_{feed}$, *n*, *n’*, *m*_*pro*_ and *M*_*w,*__*feed*_ refer to the price of feedstock ($ kg^−1^), the number of transferred electrons per turnover of product, molar ratio of feedstock to product, moles of product and molecular weight of feedstock. Considering feedstock conversion ratio (*η*_*conv*_), *C*_*feed*_ is obtained:

(Equation 13) $$C_{feed}=\frac{C_{feed,0}}{\eta_{conv}}$$

If the overall turnover number of catalysts is fixed for specific cathodic reaction, the amount of cathodic catalyst should be proportional to *m*_*pro*_. Hence, *C*_*catal*_ can be denoted as $\eta_{catal}C_{feed,\;0}$, where $\eta_{catal}$ varies with the cathodic reactions. Previous work revealed that in the chemical synthesis from CO_2_ and CO, $\eta_{catal}$ of 10–80% was required with noble metal catalysts, while the value can be significantly reduced to the order of 1% with living microbial catalysts (Christodoulou and Velasquez-Orta, 2016). Since some enzymes are not commercially available in present study, the cost for the lab scale production of enzyme is used first to estimate the cost for commercial ones. In a lab scale, enzyme cost of 1 kg substrate is about $12.7–14.4 (Meng et al., 2020; Han et al., 2020). Thus, we assume that the cost of enzyme in a lab scale in general is $20/kg substrate (higher than the value in literatures) and therefore commercially is $10/kg substrate. And the total turnover number of the enzyme is estimated as 1000 (which is indeed not a high value). On this assumption, a rough η_catal_ (ratio of cathodic catalyst cost to feedstock) range of 0.05–0.8 was adopted. η_catal_ = 0.8 is a cost based on small scale purification of enzymes with low cost substrates and η_catal_ = 0.05 is a cost on the assumption that they are commercially produced in the future and for catalyzing high cost substrates. For the product separation, the cost for separation unit and additional energetic consumption are considered. The former is already included in term *X*, while the latter (*C*_*sep*_) can be estimated by calculating the minimum work for separation (*W*_*min*_, kJ mol^−1^) with the empirical second-law efficiency values (*η*_*sec*_) (House et al., 2011; Verma et al., 2019). With assumed molar ratio of separated product *η*_*sep*_, *C*_*sep*_ and term *Y* can be obtained:

(Equation 14) $$C_{sep}=\frac{\eta_{sep}\times p_{elec}\times W_{min}\times m_{pro}}{\eta_{sec}}$$

(Equation 15) $$\begin{array}{l} Y=C_{pstat}+\frac{C_{feed,\;0}}{\eta_{conv}}+\eta_{catal}C_{feed,\;0}+\frac{p_{elec}\times W_{min}\times m_{pro}}{\eta_{sec}}\\ \;=p_{elec}\times U_{cell}\times Q+\left(\frac{1}{\eta_{conv}}+\eta_{catal}\right)\times p_{feed}\times n^{\prime }\times M_{w,\;feed}\\ \;\times \frac{Q\times FE_{c}}{n\times F}+\frac{\eta_{sep}\times p_{elec}\times W_{min}\times m_{pro}}{\eta_{sec}}\times \frac{Q\times FE_{c}}{n\times F} \end{array}$$

Every sub-term in Equation 13 is proportional to *Q*. Besides that, although a series of parameters are involved in Equation 13, they are constant for specific cathodic reaction. Therefore, the *Y* value for unit transferred electron can be obtained.

The net profits and prerequisite for cathodic reaction

Taking the value of product (*V*_*p*_) into consideration, the net profit can be denoted as:

(Equation 16) *net profits = V*_*p*_*– Y – X*

Where, *V*_*p*_ *– Y* can be viewed as the net added-value from a specific cathodic reaction. Therefore, *V*_*p*_ *– Y* > *X* is prerequisite for a proper cathodic reaction, meaning that net added-value for a specific cathodic reaction is supposed to cover the basic cost in electrochemical catalysis when the wastewater is refined in the bioelectrochemical system.

### Quantification and statistical analysis

Figures represent averaged or representative results of multiple independent experiments. Analyses and plots were performed with Origin.

## Data Availability

• All data reported in this paper will be shared by the lead contact upon request. • This paper does not report original code. • Any additional information required to reanalyze the data reported in this paper is available from the lead contact upon request.
