# Supplementary material for: Wastewater-powered high-value chemical synthesis in a hybrid bioelectrochemical system
Source: iScience. 2021 Nov 6;24(12):103401. doi: 10.1016/j.isci.2021.103401 (PMC8605441; doi:10.1016/j.isci.2021.103401)
Supplement: Document S1. Figures S1–S15 and Tables S1–S5 [file mmc1.pdf]

iScience, Volume *24*

Supplemental information

Wastewater-powered high-value chemical synthesis in a hybrid bioelectrochemical system

Ranran Wu, Yang-Yang Yu, Yuanming Wang, Yan-Zhai Wang, Haiyan Song, Chunling Ma, Ge Qu, Chun You, Zhoutong Sun, Wuyuan Zhang, Aitao Li, Chang Ming Li, Yang-Chun Yong, and Zhiguang Zhu

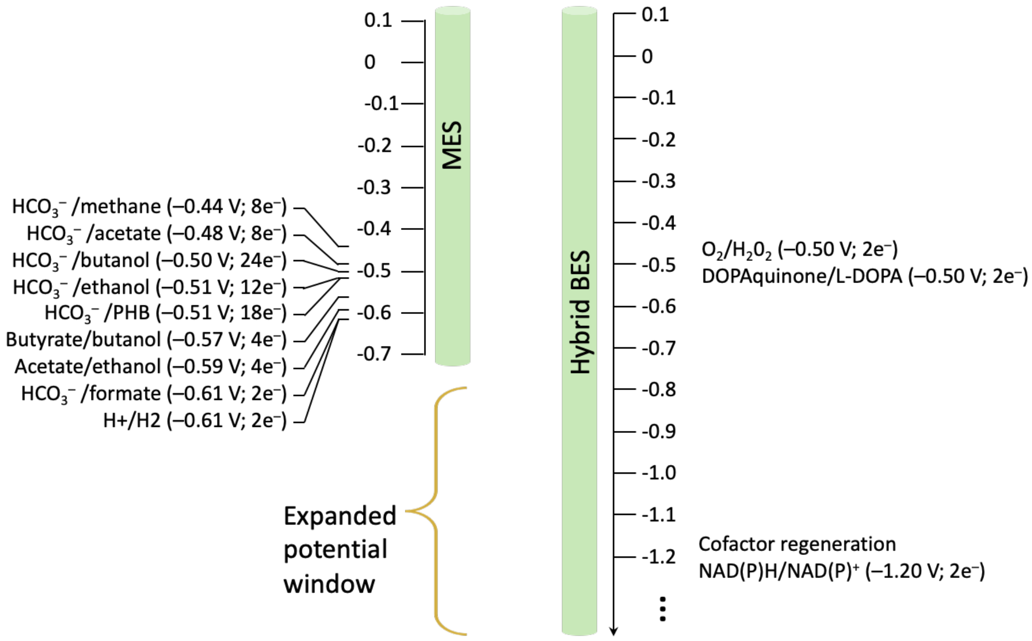

Figure S1. Reduction potentials of several electrosynthesis reactions at the cathode. Related to Introduction. *The electrode potentials of all electron acceptors are referenced from a previous study(Rabaey and Rozendal, 2010). All the potentials in this figure were versus Ag/AgCl.

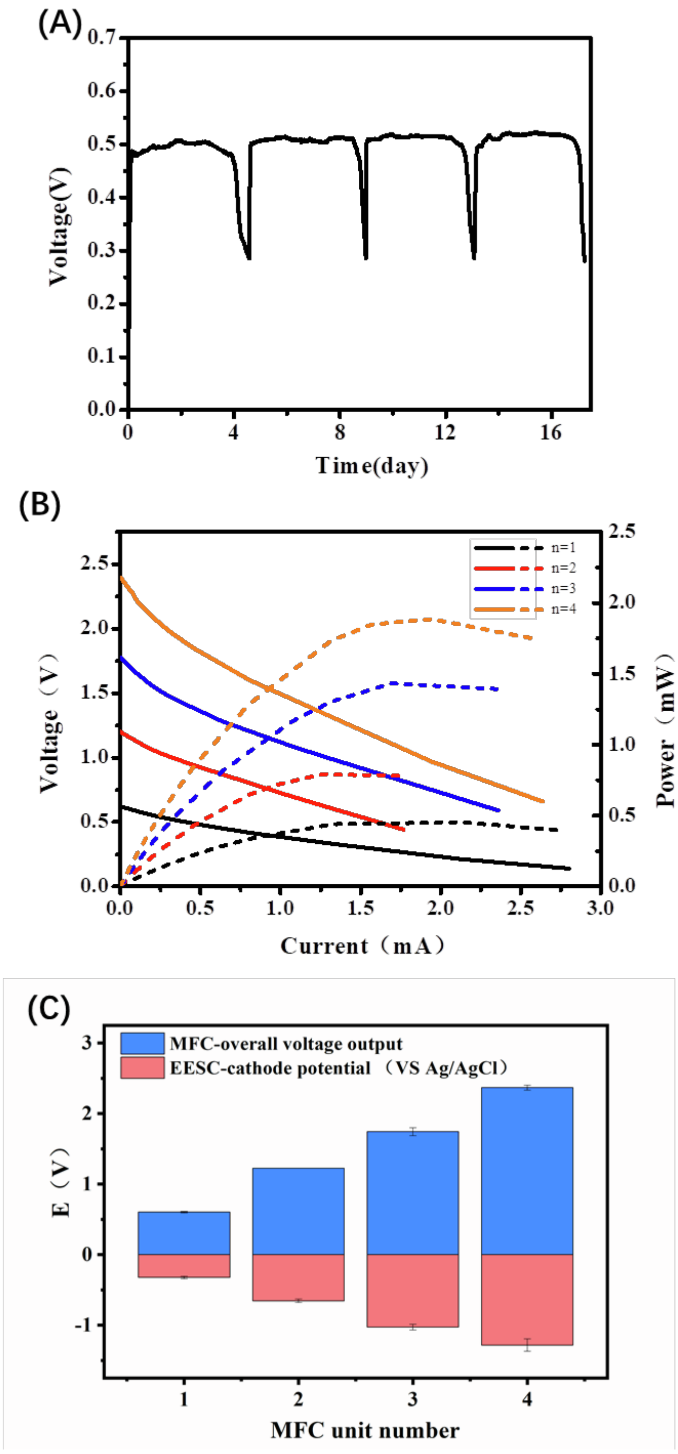

Figure S2. Performance of air-cathode MFC stacks connected in series. Related to Figure 1. (A) Voltage of a single air-cathode MFC running with a 2000 Ω external resistance. (B) Polarization curves of air-cathode MFC stacks connected in series. (C) Cathodic potentials of the EESC (*vs.* Ag/AgCl) and output voltage of the MFC stack connected in series with different unit numbers.

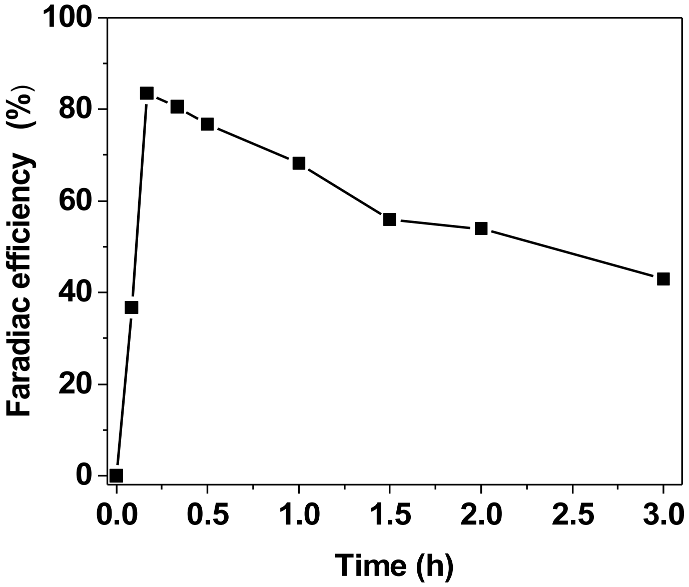

Figure S3. Faradaic efficiencies of the CF electrode during H_2O_2 production. Related to Figure 2.

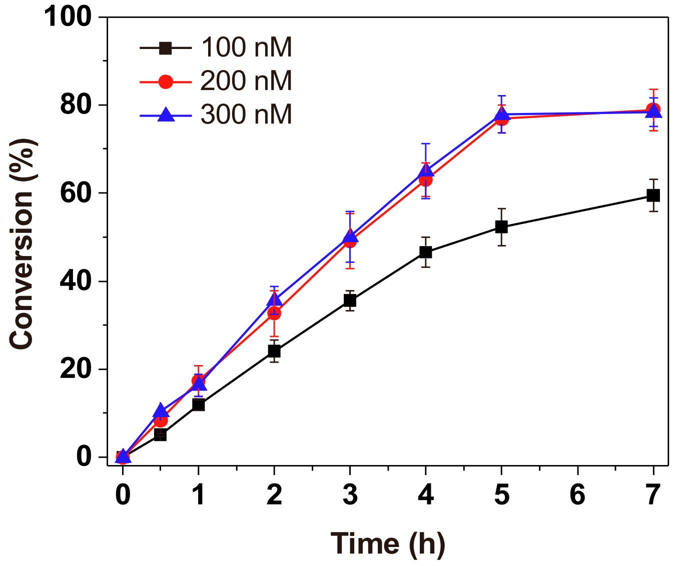

Figure S4. Conversion of 1b using different enzyme loadings. Related to Figure 2. 100 nM (black line), 200 nM (red line) and 300 nM (blue line) of enzymes were used respectively. Initially, 5 mM **1a** and 20 mM KBr were added to a citrate buffer (0.1 M, pH 5.0) with 40% ethanol as a cosolvent.

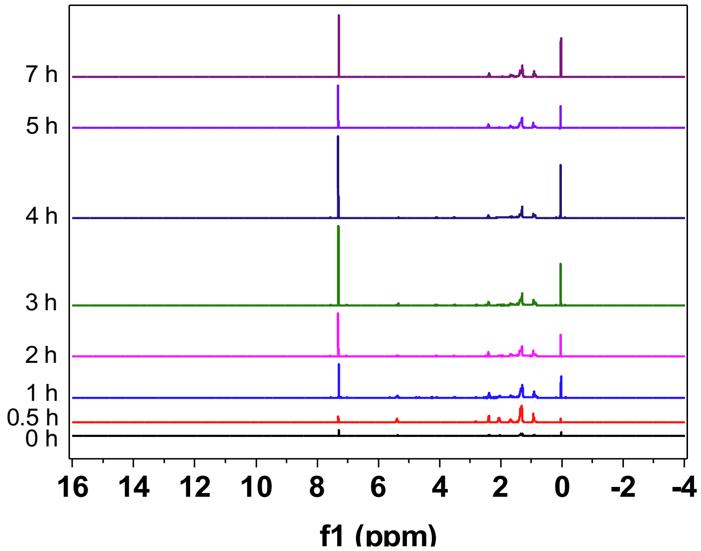

Figure S5. Raw data for nuclear magnetic resonance (NMR) spectra of the 1b conversion by the hybrid BES after reacting for 0 h, 0.5 h, 1 h, 2 h, 3 h, 4 h, 5 h and 7 h. Related to Figure 2.A single MFC was used as the power source with a potential of −0.5 V at the EESC cathode and 5 mM **1a** was added. 10-Bromo-9-hydroxyoctadecanoic acid and 9-bromo-10-hydroxyoctadecanoic acid were identified by crude ^{1}H NMR spectra. ^{1}H NMR (400 MHz, $CDCl_3$) δ: 0.85 (3H, m), 1.16-1.87 (26H, m), 2.31-2.36 (2H, m), 3.33-3.35 (1H, m), 4.03-4.05 (1H, m).

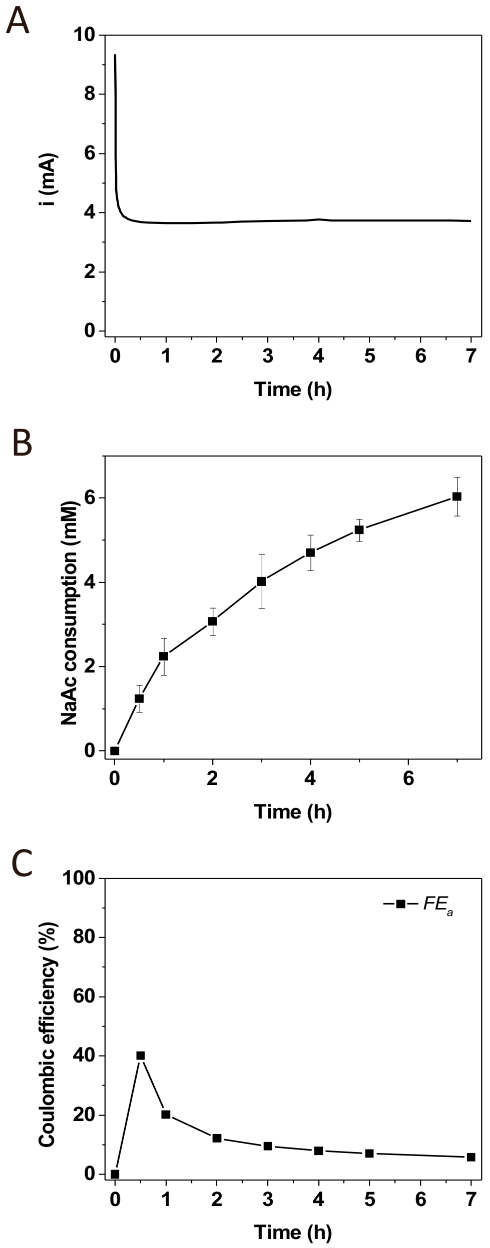

Figure S6. Faradaic efficiencies of the anode. Related to Figure 2. (A) Current in the circuit detected by a multimeter connected in series, (B) consumption of sodium acetate in both MFC anode and EESC anode, and (C) faradaic efficiencies of the anode during halofunctionalized oleic acid production in thee hybrid BES. A single MFC was used as the power source. 1 mL sodium acetate (1 M) was added into each anode, while 5 mM oleic acid, 20 mM KBr and 200 nM CiVCPO were added into the EESC cathode at 30°C.

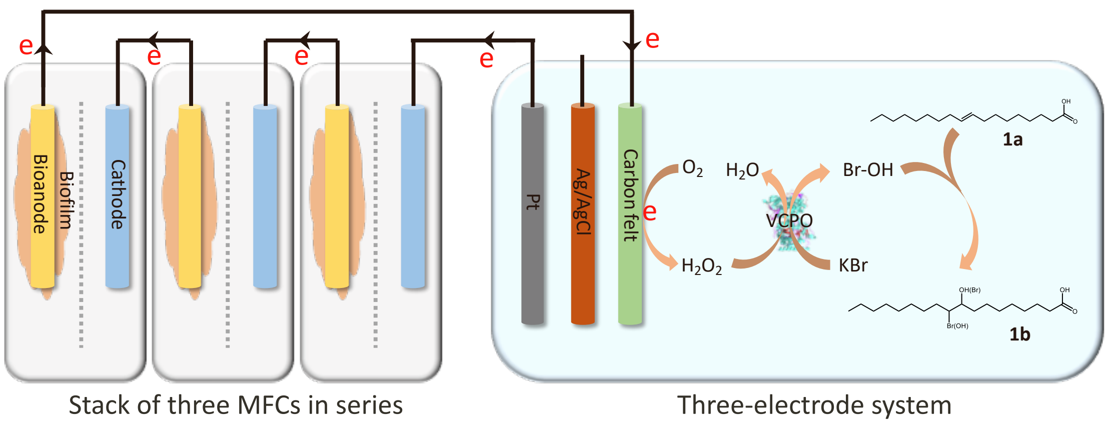

Figure S7. Schematic of the electroenzymatic synthesis of halofunctionalized oleic acid in a microbe-enzyme hybrid BES using a three-electrode system EESC. Related to Figure 2.

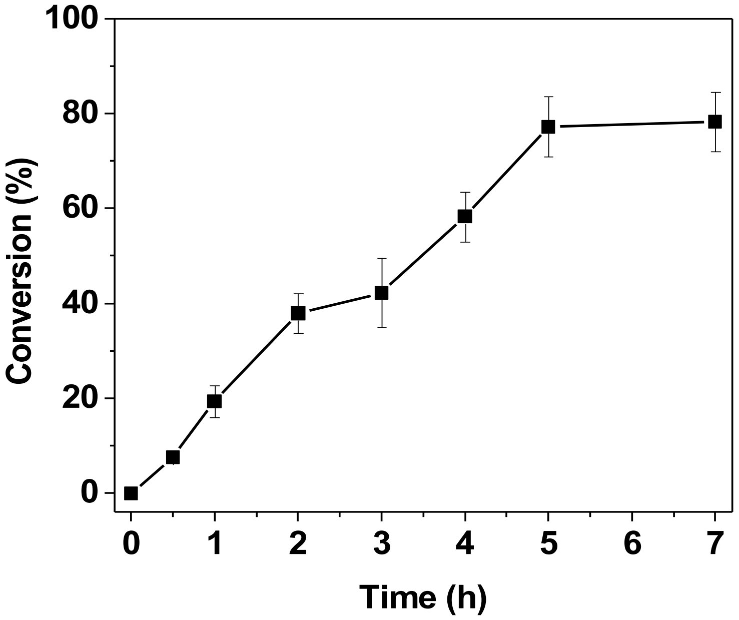

Figure S8. 1b yeild under a potential of −0.5 V. Related to Figure 3. A three-electrode system EESC powered by a three-MFC stack at 30°C was used and citrate buffer (0.1 M, pH 5.0) with 40% ethonal served as co-solvent. 200 nM CiVCPO, 5 mM **1a** and 20 mM KBr were added.

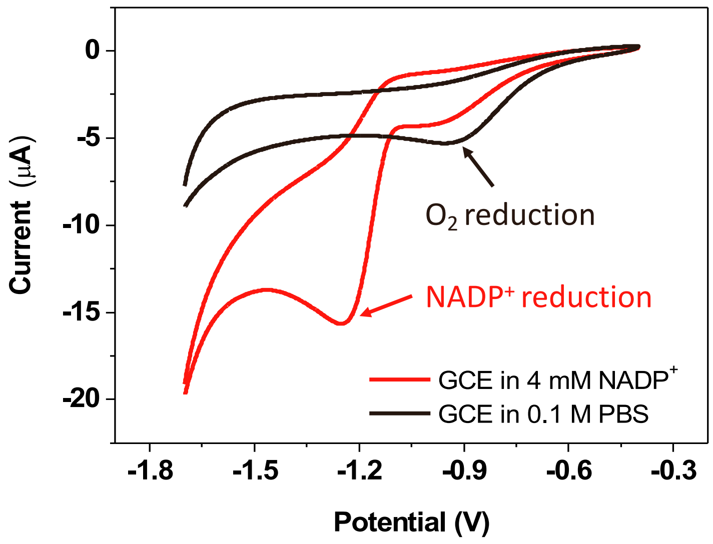

Figure S9. Cyclic voltammogram of $NADP^+$ on a GCE. Related to Figure 3. A three-electrode system with glassy carbon electrode (GCE, Φ3 mm), Ag/AgCl and Pt sheet (1×1 cm^2) served as working electrode, reference electrode and counter electrode was used in 5 mL phosphate buffer (pH 7.0, 0.1 M). 4 mM $NADP^+$ was added. Scan rate: 10 mV s^{-1}.

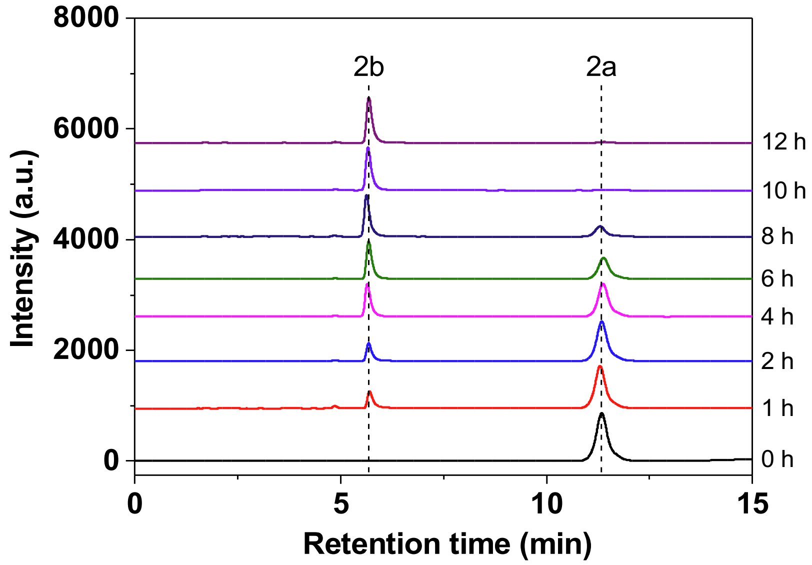

Figure S10. Raw data for HPLC profile of 2b conversion by the hybrid BES after reacting for 0 h, 1 h, 2 h, 4 h, 6 h, 8 h, 10 h and 12 h. Related to Figure 3. A stack of three MFCs was used as the power source. Initially, 2 mM NADPH, 20 mM CPMK and crude TbSADH (~100 mg protein) were present. Representative peaks of **2a** and **2b** were labeled.

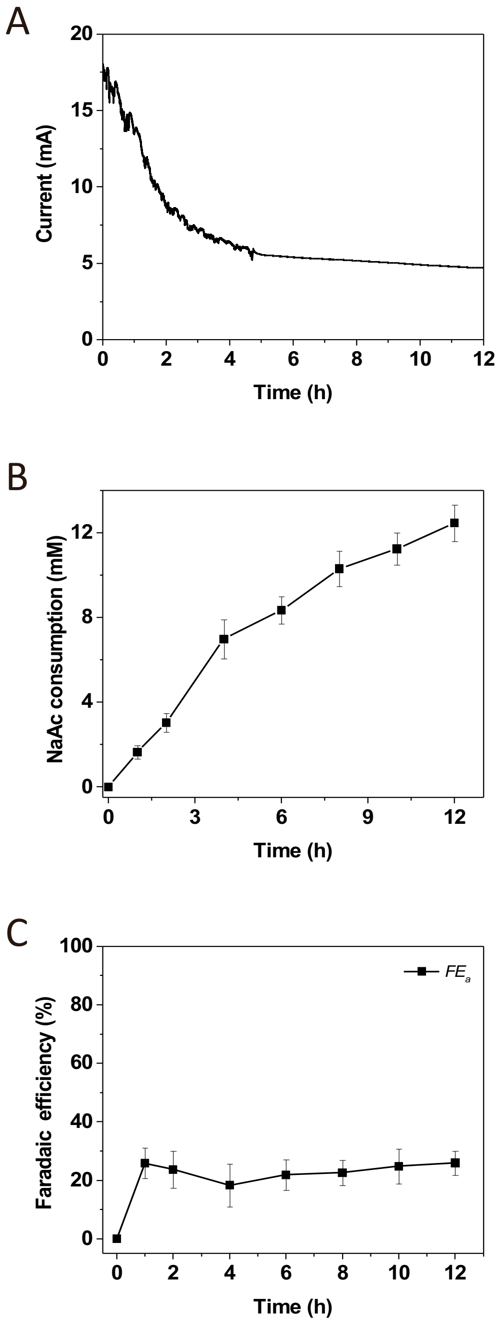

Figure S11. Faradaic efficiencies of the anode. Related to Figure 3. (A) Current in the circuit detected by a multimeter connected in series, (B) total consumption of sodium acetate in both MFC anode and EESC anode, and (C) faradaic efficiencies of the anode during CPMA synthesis in thee hybrid BES. Series-stacked three MFCs were used as the power source. 1 mL sodium acetate (1 M) was added into each anode, while 20 mM CPMK, 2 mM NADPH and crude TbSADH (~100 mg protein) were added into the EESC cathode at 30°C.

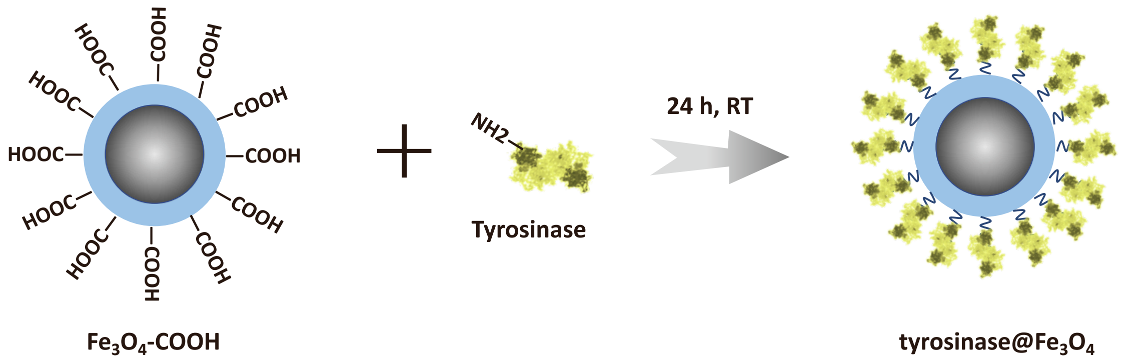

Figure S12. Schematic of tyrosinase immobilization using carbonylated magnetic Fe_3O_4 nanoparticles via the amide bond. Related to Figure 4.

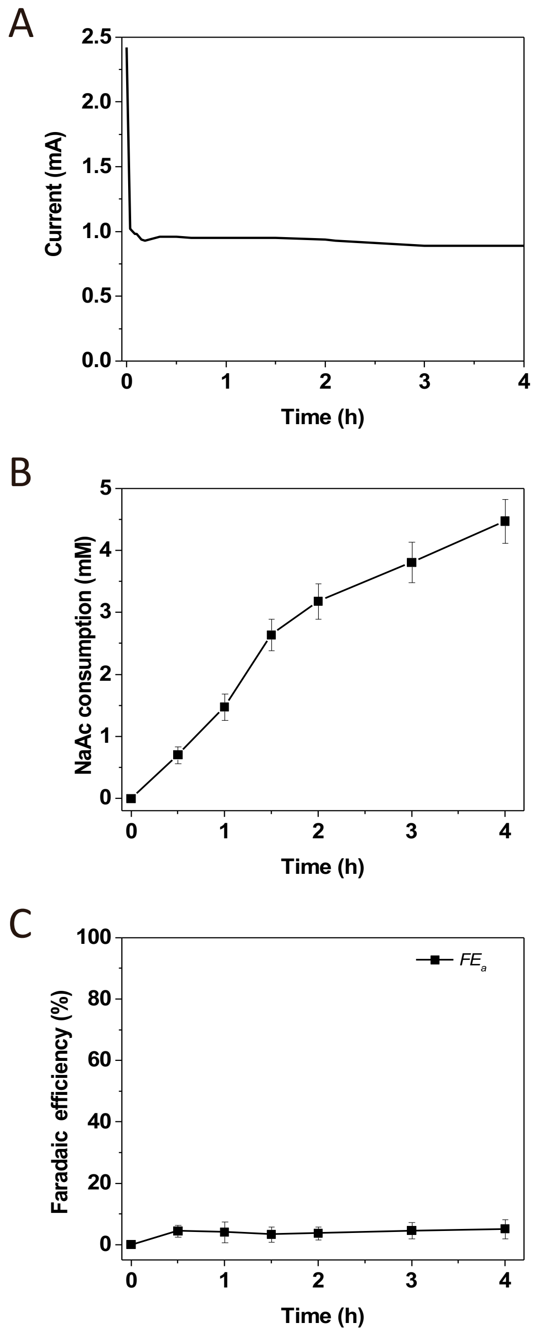

Figure S13. Faradaic efficiencies of the anode. Related to Figure 4. (A) Current in the circuit detected by a multimeter connected in series, (B) total consumption of sodium acetate in both MFC anode and EESC anode, and (C) faradaic efficiencies of the anode during L-DOPA synthesis using the hybrid BES. A single MFC was used as the power source. 1 mL sodium acetate (1 M) was added into each anode, while 1 mM L-tyrosine and tyrosinase@Fe_3O_4 with 0.25 mg tyrosinase were added into the EESC cathode.

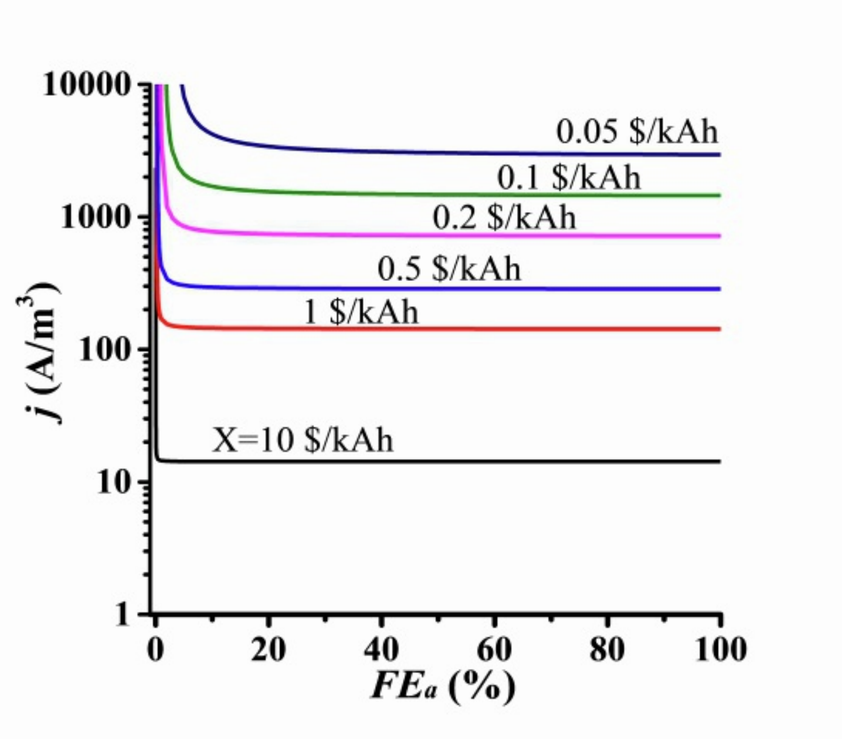

Figure S14. The simulated result of fixed cost *X*. Related to Figure 5. Basic parameters: C_o=2400 \$ m^{-3}, ΔCOD=500 g m^{-3}, FE_a=60%, V_R=1 m^3, V_a=0.5 m^3, t=4380 h y^{-1}, U^{Θ}=0.2 V; Term X consists of cost for investment, maintenance and energy for pumping, which can be viewed as the basic cost for electrochemical catalysis and independent with the cathodic reaction.

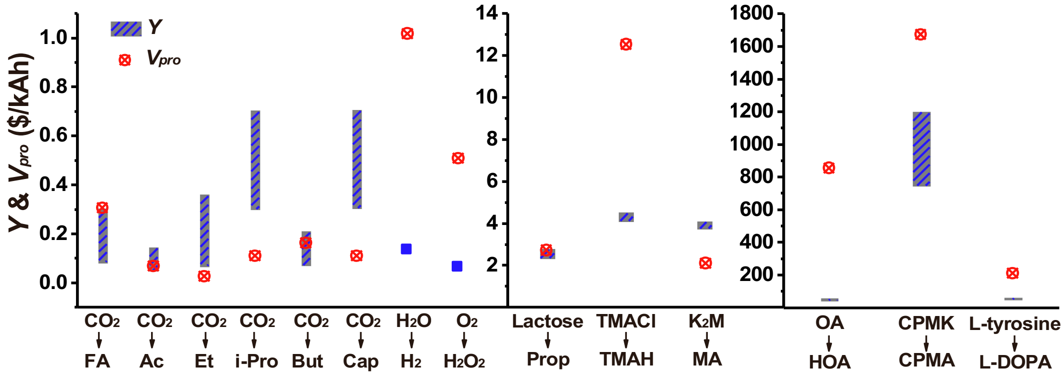

Figure S15. The simulated variable costs *Y* and values of typical products synthesized at bioelectrochemical systems. Related to Figure 5. *Y* refers to the additional cost of a specific cathodic reaction including energy for potential control, cathode catalyst, feedstock and energy for separation. Parameters for calculating Term Y are listed in Table S3. *Y* varies with the cathodic conditions such as feedstock sources (e.g. commercial CO_2 *vs.* recycled CO_2 from bioanode) and cost of cathodic catalysts (current *vs.* future). Abbreviations: FA (formic acid), Ac (acetic acid), Et (ethanol), i-Pro (isopropanol), But (butyrate), Cap (caproate), Prop (propionate), TMAH (Tetramethylammonium hydroxide), TMACl (tetramethylammonium chloride), MA (malic acid), K_2M (potassium malate), OA (oleic acid), HOA (hydroxy/halo-functionalized oleic acid oleic acid), CPMA ((4-chlorophenyl)-(pyridin-2-yl)methanol), CPMK (ketone 4-chlorophenyl)(pyridin-2-yl)methanone), and L-DOPA (l-3,4-dihydroxyphenylalanine).

Table S1. The ions in the dual or single electrochemical synthesis cell. Related to Results and discussion.

Ion		EESC cathode with open circuit	EESC cathode with a MFC as the power source	Three-electrode synthesis cell with a three-MFC stack as the power source
Anion	PO_4^{3-}	＜LOQ (200 ppm[a])	279	＜LOQ (200 ppm)
	NO_3^-	＜LOQ (150 ppm)	＜LOQ (150 ppm)	＜LOQ (150 ppm)
	$CH3COO^-$	＜LOQ (50 ppm)	＜LOQ (50 ppm)	＜LOQ (50 ppm)
Cation	Fe^{2+}/Fe^{3+}	＜LOQ (40 ppm)	＜LOQ (40 ppm)	＜LOQ (40 ppm)
	Cu^{2+}	＜LOQ (40 ppm)	＜LOQ (40 ppm)	＜LOQ (40 ppm)
	Zn^{2+}	＜LOQ (40 ppm)	＜LOQ (40 ppm)	＜LOQ (40 ppm)
	Pb^{2+}	＜LOQ (0.2 ppm)	＜LOQ (0.2 ppm)	＜LOQ (0.2 ppm)

[a]ppm=mg kg^{-1}.

Table S2. The terms and parameters in modeling term *X* and *Y*. Related to Figure 5.

Term	Definition	Formula	Unit	Values	Ref.
ΔCOD	consumed organic substrate in wastewater after anode treatment		g m^{-3}	*100-10,000*	(Jadhav and Ghangrekar, 2009; Mohan et al., 2009)
V_a	anodic volume		m^3	*depends*	
τ	anodic hydraulic retention time		h	*depends*	
t	system working time		h year^{-1}	4380	
V	the total volume of treated wastewater	$\frac{V_a}{\tau} \times t$	m^3	*depends*	
F	Faraday constant		C mol^{-1}	96485	
j	current density		A m^{-3}	10-10,000	
M_{w,O_2}	molecular weight of oxygen		g mol^{-1}	32	
FE_a	anode faradaic efficiency, defined as the ratio of electron harvested at anode to the theoretical value in *ΔCOD*	$\frac{M_{w,O_2} \times \tau \int_0^t j(t)dt}{4 \times \Delta COD \times F \times t}$	N. D	10-80%	(Lefebvre et al., 2011)
Q	total charge transferred across external circuit	$\frac{4 \times \Delta COD \times F \times FE_a}{M_{w,o2}} \times V$	C	*depends*	
m_{pro}	moles of product		mol	*depends*	
n	number of transferred electrons per turnover of product		N. D	*depends*	
FE_c	cathode faradic efficiency, defined as the ratio of electron for target chemical production to the theoretical value across external circuit	$\frac{n \times m_{pro} \times F}{Q}$	N. D	20-100%	(Zhen et al., 2017)
U^o	standard cell voltage for the overall reaction in anode and cathode		V	*depends*	

U_{cell}	applied potential between anode and cathode		V	*depends*	
EE	energetic efficiency	$\frac{U^o \times FE_c}{U_{cell}}$	N. D	30-80%	(Claassens et al., 2019)
p_{elec}	price of electricity		\$ kWh^{-1}	0.1	(House et al., 2011)
P_{pump}	power for pumping wastewater		kWh m^{-3}	0.027	(Dong et al., 2015)
V_{cell}	total volume of reactor		m^3	*depends*	
p_{cell}	price of volumetric reactor		\$ m^{-3}	1200	(Escapa et al., 2012; He et al., 2019)
$P_{a\text{-}equip}$	price of accessory equipment	1:1 to p_{cell}	\$ m^{-3}	1200	(Christodoulou and Velasquez-Orta, 2016)
C_0	total investment	$V_{cell} \times (p_{cell} + P_{a\text{-}equip})$	\$	*depends*	
m	assumed years of operation/loan		y	20	
r	annual interest rate		N. D	5%	
C_{cap}	annual capital cost	$C_0 \frac{(1+r)^m \times r}{(1+r)^m - 1}$	\$ y^{-1}	*depends*	(Claassens *et al.*, 2019)
C_m	annual maintenance cost	5% × C_0	\$ y^{-1}	*depends*	
C_{pump}	energetic cost for wastewater pumping	$p_{elec} \times P_{pump} \times V$	\$	*depends*	
C_{pstat}	energetic cost for potential control	$p_{elec} \times U_{cell} \times Q$	\$	*depends*	(Claassens *et al.*, 2019)
p_{feed}	price of feedstock		\$ kg^{-1}	*depends*	
n'	molar ratio of feedstock to product		N. D	*depends*	
η_{conv}	molar ratio of feedstock transformed to target product *vs.* theoretical value		N. D	*depends*	
$M_{w,pro}$	molecular weight of target product		g mol^{-1}	*depends*	
$C_{feed,0}$	stoichiometric cost for feedstock	$p_{feed} \times n' \times m_{pro} \times M_{w,feed}$	\$	*depends*	

C_{feed}	cost for feedstock	$\frac{C_{feed,0}}{\eta_{conv}}$	\$	*depends*	
η_{catal}	ratio of cathodic catalyst cost to feedstock		N. D	0.1-80%	(Christodoulou and Velasquez-Orta, 2016)
C_{catal}	cost for cathode catalyst for volumetric wastewater	$\eta_{catal} \times C_{feed,0}$	\$	*depends*	
W_{min}	minimum work for separation		kJ mol^{-1}	10-40	(House *et al.*, 2011)
η_{sec}	empirical second-law efficiency values		N. D	2-50%	(House *et al.*, 2011)
η_{sep}	assumed molar ratio of separated product *vs.* m_{pro}			90%	
C_{sep}	cost for energetic consumption in separation	$\frac{\eta_{sep} \times p_{elec} \times W_{min} \times m_{pro}}{\eta_{sec}}$	\$	*depends*	
p_p	price of product		\$ kg^{-1}	*depends*	
V_p	value of product	$p_p \times \eta_{sep} m_{pro \times} M_{w,pro}$	\$	*depends*	

Table S3. Simulated basic cost (X) for bioelectrochemical refinery of wastewater. Related to Figure 5. The rest parameters are: C_o=2400 \$ m^{-3}, ΔCOD=500 g m^{-3}, FE_a=60%, V_R=1 m^3, V_a=0.5 m^3, t=4380 h y^{-1}.

	j=100 A m^{-3} FE_a=10%	j=100 A m^{-3} FE_a =80%	j=10,000 A m^{-3} FE_a =10%	j=10,000 A m^{-3} FE_a =80%
X (\$ kAh^{-1})	1.4408	1.426713	0.030347	0.01626
$C_{cap}+C_m$%	98.9	99.8	45.9	87.6
C_{pump}%	1.1	0.2	55.1	12.4

Table S4. The parameters for simulation of *Y* and V_{pro}. for formic acid, acetic acid, ethanol and butanol production produced from bioelectrochemical refinery of 1 m^3 wastewater. Related to Figure 5.

Raw material	CO_2	CO_2	CO_2	CO_2	CO_2	CO_2	H_2O	O_2	lactose	TMACl	K_2M	OA	CPMK	L-tyrosine
Product	formic acid	acetic acid	ethanol	isopropanol	butyrate	caproate	H_2	H_2O_2	propionate	TMAH	malic acid	HOA	CPMA	L-dopa
p_{elec} **($ kWh^{-1})**	0.1	0.1	0.1	0.1	0.1	0.1	0.1	0.1	0.1	0.1	0.1	0.1	0.1	0.1
FEc	0.77	0.8	0.6	0.137	0.7	0.112	1	0.7	1.01	0.55	0.59	0.91	1.02	1.02
U_{cell}	0	0.3	0.5	0.8	0.6	0.65	0.4	0	0.35	1	1	0	0	0
η_{conv} [a]	0.9	0.9	0.45	0.21	0.6	0.069	0.98	1	**0.8-0.99**	**0.8-0.9**	**0.8-0.9**	0.78	0.87	0.64
η_{catal} [b]	0.05	0.01	0.01	0.01	0.01	0.01	0.01	0.01	0.01	0.05	0.05	**0.05-0.8**	**0.05-0.8**	**0.05-0.8**
p_{feed} **($ kg^{-1})** [c]	**0.04-0.2**	**0-0.2**	**0-0.2**	**0-0.2**	**0-0.2**	**0-0.2**	0	0.002	1.31	3	1.8	6	61.7	9.7
n	2	8	12	18	20	20	2	2	2	2	2	2	2	2
n'	1	2	2	3	4	4	1	1	0.25	1	1	1	1	1
$M_{w\,feed}$	44.01	44.01	44.01	44.01	44.01	44.01	18.02	34.01	342.26	109.6	154.16	282.46	217.65	146.19
p_{sep} **($ mol^{-1})** [d]	0.011	0.011	0.011	0.011	0.011	0.011	0.011	0.011	0.011	0.011	0.011	0.011	0.011	0.011
η_{sep}	0.9	0.9	0.9	0.9	0.9	0.9	0.9	0.9	0.9	0.9	0.9	0.9	0.9	0.9
$M_{w,pro}$	46.03	60.08	46.07	60.06	88.11	116.16	2.02	32.00	88.06	91.05	116.07	379.38	219.65	162.19
$p_{product}$ **($ kg^{-1})**	0.5	0.4	0.8	1.27	1.5	6.24	30.0	1.25 (35%)	1.8	14.9	1.8	149.4	223.7	79.1
Reference	***(Zhao et al., 2012)***	***(Xiang et al., 2017)***	***(Srikanth et al., 2018)***	***(Arends et al., 2017)***	***(Batlle-Vilanova et al., 2017)***	***(Jourdin et al., 2018)***	***(Zhang et al., 2010)***	***(Fu et al., 2010)***	***(Schuppert et al., 1992)***	***(Liu et al., 2014)***	***(Liu et al., 2015)***	***This study***	***This study***	***This study***

The U_{cell} was recalculated in the case that cathodic potential was controlled in three-electrode system and appeared as (V *vs.* standard electrode). Presumed anodic potential: -0.2 V *vs.* SHE.

[a] In case the η_{conv} was not provided in the original work, presumed value of 90% was adopted. η_{conv} for propionate, TMAH or malic acid production was treated as variable.

[b] η_{catal} for HOA, CPMA or L-DOPA, was treated as variable. Specific enzymes were used in this study but they are not commercially available so far. Hence, they are much more expensive than bioelectrochemical systems with electroactive bacteria as the cathodic catalyst. But this cost would be significantly reduced if they are commercialized in the future. So the adopted η_{catal} range (0.05-0.8) is only a rough estimation based on the assumption that the cost of enzyme in lab scale is \$20 /$kg_{substate}$ (higher than the value in literatures(Han et al., 2020; Meng et al., 2020)) and commercially is \$10 /$kg_{substate}$, and the total turnover number of the enzyme is 1000 (which is very low and result in a higher cost for each enzyme molecule). η_{catal}= 0.8 is a cost based on small scale purification of enzymes with low cost substrates and η_{catal}= 0.05 is a cost on the assumption that they are commercial in the future catalyzing high cost substrates.

[c] In CO_2 reduction cathode, p_{feed} varied with the source of CO_2. If only commercial CO_2 was used, a value of 0.2 \$ kg^{-1} was adopted, whereas feeding the cathode with recycled CO_2 in anodic chamber significantly reduced its cost. Theoretically, the CO_2 released from anodic COD oxidation can completely cover cathodic CO_2 consumption except that formic acid is the product. The “zero” value of p_{feed} in these cases does not mean that cost for feedstock is completely avoided since the energy cost for CO_2 recycling and auxiliary materials are not considered, but it can be regarded as a theoretical low limit.

[d] For simplification, W_{min} and η_{sec} were assumed to be 20 kJ mol^{-1} and 5%. As a result, p_{sep} for unit mole product was treated as constant. The actual p_{sep} can vary with the complexity of product mixture and choice of specific technique process. For example, in the case of acetic acid production, the above W_{min} and η_{sec} give an energetic consumption of 1.9 kWh kg^{-1} product, which is close to the reported value (2.5 kWh kg^{-1})(Galitsky et al., 2008). Therefore, such simplification is beneficial for the fast estimation of the overall cost for specific product.

Table S5. Comparison of HOA, CPMA and L-DOPA production via different synthetic method. Related to Figure 5.

HOA

Substrate	Concentration (mM)	Enzyme	H_2O_2 source	Additive cost ($ kg^{-1})	Conversion (%)	Ref
OA	20	CiVCPO	H_2O_2	0.8~1.5	62	(Dong et al., 2017)
OA	5	CiVCPO	Electrode	N/N	78.93 ± 4.6	This work

CPMA

Substrate	Concentration (g L^{-1})	Organism/ Enzyme	NAD(P)H regeneration	Additive	Additive cost ($ kg^{-1})	Conversion (%)	Ref
CPMK	6	Kluyveromyces sp.	GDH	Glucose	0.7~1.5	92.1	(Ni et al., 2012)
CPMK	2	*Cryptococcus sp.*	GDH	Glucose	0.7~1.5	92	(Xu et al., 2017)
Benzoylpyridine derivatives	1	ketoreductase	GDH	Glucose	0.7~1.5	98	(Truppo et al., 2007)
CPMK	6	TbSADH	TbSADH	Isopropanol	0.8~1.3	>99	(Liu et al., 2019)
CPMK	4.4	TbSADH	Electrode	N/N	N/N	86.8 ± 3.3	This work

L-DOPA

Substrate	Concentration (mM)	Organism/ Enzyme	Energy input	Reductant cost ($)	Productivity	Ref
L-tyrosine	2.5	Tyrosinase	Ascorbic acid	4~7 kg^{-1}	110 mg L^{-1} h^{-1}	(Ates et al., 2007)
L-tyrosine	1–4	Tyrosinase	Ascorbic acid	4~7 kg^{-1}		(Tuncagil et al., 2009)
L-tyrosine	0.1–4.5	Recombinant *E. coli* harboring tyrosine phenol-lyase	N/N	N/N	110 g L^{-1}	(Zheng et al., 2018)
L-tyrosine	1	Tyrosinase	–0.53 V vs. Ag/AgCl	0.14~0.17 kW·h	47.27 mg L^{-1} h^{-1}	(Min et al., 2010)
L-tyrosine	0.1–1.0	Tyrosinase	–0.53 V vs. Ag/AgCl	0.14~0.17 kW·h	15.3 g L^{-1} h^{-1}	(Min et al., 2013)
L-tyrosine	1	Tyrosinase	Glucose	0.7~1.5 kg^{-1}	118.3 mg L^{-1} h^{-1}	(Wu and Zhu, 2018)
L-tyrosine	1	Tyrosinase	Wastewater	N/N	84.1 mg L^{-1} h^{-1}	This work
